# Whole plastid transcriptomes reveal abundant RNA editing sites and differential editing status in *Phalaenopsis aphrodite* subsp. *formosana*

**DOI:** 10.1186/s40529-017-0193-7

**Published:** 2017-09-16

**Authors:** Ting-Chieh Chen, Yu-Chang Liu, Xuewen Wang, Chi-Hsuan Wu, Chih-Hao Huang, Ching-Chun Chang

**Affiliations:** 1 Institute of Biotechnology, National Cheng Kung University, Tainan, 701 Taiwan; 2 Department of Biotechnology and Bioindustry Sciences, National Cheng Kung University, Tainan, 701 Taiwan; 3 Department of Genetics, University of Georgia, Athens, GA 30602 USA

**Keywords:** Plastids, Plastid gene expression, RNA editing, Moth orchids, *Phalaenopsis aphrodite*

## Abstract

**Background:**

RNA editing is a process of post-transcriptional level of gene regulation by nucleotide modification. Previously, the chloroplast DNA of Taiwan endemic moth orchid, *P. aphrodite* subsp. *formosana* was determined, and 44 RNA editing sites were identified from 24 plastid protein-coding transcripts of leaf tissue via RT-PCR and then conventional Sanger sequencing. However, the RNA editing status of whole-plastid transcripts in leaf and other distinct tissue types in moth orchids has not been addressed. To sensitively and extensively examine the plastid RNA editing status of moth orchid, RNA-Seq was used to investigate the editing status of whole-plastid transcripts from leaf and floral tissues by mapping the sequence reads to the corresponding cpDNA template. With the threshold of at least 5% C-to-U or U-to-C conversion events observed in sequence reads considered as RNA editing sites.

**Results:**

In total, 137 edits with 126 C-to-U and 11 U-to-C conversions, including 93 newly discovered edits, were identified in plastid transcripts, representing an average of 0.09% of the nucleotides examined in moth orchid. Overall, 110 and 106 edits were present in leaf and floral tissues, respectively, with 79 edits in common. As well, 79 edits were involved in protein-coding transcripts, and the 58 nucleotide conversions caused the non-synonymous substitution. At least 32 edits showed significant (≧20%) differential editing between leaf and floral tissues. Finally, RNA editing in *trnM* is required for the formation of a standard clover-leaf structure.

**Conclusions:**

We identified 137 edits in plastid transcripts of moth orchid, the highest number reported so far in monocots. The consequence of RNA editing in protein-coding transcripts mainly cause the amino acid change and tend to increase the hydrophobicity as well as conservation among plant phylogeny. RNA editing occurred in non-protein-coding transcripts such as tRNA, introns and untranslated regulatory regions could affect the formation and stability of secondary structure, which might play an important role in the regulation of gene expression. Furthermore, some unidentified tissue-specific factors might be required for regulating RNA editing in moth orchid.

**Electronic supplementary material:**

The online version of this article (doi:10.1186/s40529-017-0193-7) contains supplementary material, which is available to authorized users.

## Background

RNA editing represents a process of post-transcriptional level of gene regulation by modification of the nucleotide sequence. Two distinct types of RNA editing are identified: insertion/deletion and conversion/substitution. In plant organelles, most of the RNA editing causes C-to-U and rarely U-to-C conversions. The consequence of RNA editing occurring at first or second codon positions can increase proteomic diversity but usually leads to the conservation of amino acid sequences among plant phylogeny. Some RNA editing events could result in the creation of translation initiation or termination codons or repair internal stop codons (Bock 2000; Fiebig et al. 2004; Wolf et al. 2004). Several events occurring in non-coding regions such as tRNA, 5′/3′ untranslated regulatory regions (UTR) or introns might affect RNA stability or splicing by modifying the secondary structure (Drescher et al. 2002; Farre et al. 2012; Zeng et al. 2007). The significance of RNA editing in regulating plant organellar gene expression has been demonstrated, with the alteration of some editing sites having deleteriously effects on plant growth, development, and fertility (Hammani and Giege 2014).

The current model for plant organellar RNA editing requires at least three components: the *cis*-acting elements recognized by trans-acting factors, subsequently recruiting the editing enzyme to catalyze the nucleotide conversion (Takenaka et al. 2012). The *cis*-elements include approximately 20–40 nucleotides (−30/+10) spanning the editing site, and the upstream 20 nucleotides are more important than downstream nucleotides (Bock 2000; Hammani and Giege 2014; Hirose and Sugiura 2001; Yagi et al. 2013); however, the *cis*-elements surrounding organellar editing sites are not generally conserved. The site-specific *cis*-elements are recognized by an editosome that consists of at least four nuclear-encoded protein factors (Sun et al. 2016). The pentatricopeptide repeat (PPR) protein is a large family with more than 400 members in most species of land plants (Barkan and Small 2014). The CRR4 protein, required for site-specific editing at the initiation codon of chloroplast *ndhD* transcripts, was the first identified PPR protein in Arabidopsis (Kotera et al. 2005). In addition, another group of small family proteins, multiple organellar RNA editing factors or RNA editing-interacting proteins (MORF/RIP), is required for plant organelle RNA editing (Bentolila et al. 2012, 2013; Takenaka et al. 2012, 2013, 2014). The MORF/RIP proteins could interact with PPR proteins and might serve as a bridge to connect PPR proteins and editing enzymes, but the actual function is not clear (Bentolila et al. 2012; Takenaka et al. 2013). Furthermore, a zinc finger protein, organelle zinc finger 1 (OZ1), directly interacts with organelle RNA recognition motif-containing protein 1 (ORRM1), which in turn could bind to RIPs, is also required for some C targets (Sun et al. 2013, 2015). The editing enzyme responsible for deamination or amination remains unidentified. Because of a highly conserved motif resembling the zinc-binding active site of nucleotide deaminases and essential for editing, the DYW motif of PPR protein was hypothesized to be responsible for the catalytic reaction but is still debated (Boussardon et al. 2014; Takenaka et al. 2014; Yagi et al. 2013; Wagoner et al. 2015).

The organellar RNA editing sites have been investigated in more than a dozen plant species by traditional Sanger methods, through sequencing cDNA and comparing with corresponding genomic DNA. In plastids of seed plants, the number of editing sites is relatively constant, 21 to 44 which account for less than 0.07% of genome (Sasaki et al. 2003, 2006; Zeng et al. 2007), and mostly occur in protein-coding transcripts. However, the number of mitochondrial editing sites is abundant, from 189 to 635, but varies among species (Grimes et al. 2014; Picardi et al. 2010; Sloan et al. 2010). The editing efficiency of plastid transcripts varies among ecotypes, tissue types, developmental stages and environmental factors (Bentolila et al. 2005; Tseng et al. 2013). Recently, by applying next-generation sequencing (NGS), organellar RNA editing sites have been identified more rapidly and economically and have been extensively examined at the whole transcriptome level (Bentolila et al. 2013; Grimes et al. 2014; Picardi et al. 2010). Besides the fast and large-scale detection of RNA editing sites, NGS technology has provided depth of coverage (DOC) per reference nucleotide and qualities of base call. It could overcome the existing limitations of standard Sanger methodology to sensitively revealing previously undiscovered tissue-specific or low-level partial editing sites. For instance, NGS revealed many new editing sites in the mitochondria of Arabidopsis and tobacco from leaf and/or floral tissues (Bentolila et al. 2013; Grimes et al. 2014; Picardi et al. 2010). In addition, re-addressing RNA editing status in the leaf chloroplasts of Arabidopsis and *pnp* mutant by NGS revealed several novel editing sites (Bentolila et al. 2013; Ruwe et al. 2013).

The Orchidaceae family contains approximately 25,000 species and represents one of the most diverse families in flowering plants (Dressler 2005). The *Phalaenopsis* (moth orchids) genus comprises approximately 66 endemic species (Christenson 2001; Lin et al. 2016). Moth orchids are among the top-traded blooming potted plants in the world, with more than 32,000 hybrids bred and registered in the Royal Horticultural Society (Lin et al. 2015a, b; Liu et al. 2016). The chloroplast DNAs (cpDNAs) of two endemic moth orchids, *P. aphrodite* subsp. *formosana* and *P. equestris*, have been determined (Chang et al. 2006; Jheng et al. 2012); 44 plastid RNA editing sites were identified from 24 protein-coding transcripts of leaf tissues in *P. aphrodite* subsp. *formosana* by conventional Sanger sequencing (Zeng et al. 2007); and 43 editing sites were bioinformatically predicted in *P. equestris* (Jheng et al. 2012). However, the RNA editing status of whole-plastid transcripts in leaf and other distinct tissue types in moth orchids has not been addressed.

In this study, we used NGS for transcriptome analysis to extensively study the RNA editing status in whole-plastid transcripts from leaf and floral tissues of *P. aphrodite* subsp. *formosana*. Our results revealed 93 new plastid edits and significantly differential editing status between the two tissue types.

## Methods

### Plant material and RNA extraction

The seedlings of 1.5-year-old *P. aphrodite* subsp. *formosana* cultivar TS97 were purchased from TaiSugar Corporation (Tainan, Taiwan). Approximately 50–100 g tissues from leaves or flowers was used to isolate plastids as described (Hrubec et al. 1985; Schwitzguebel and Siegenthaler 1984) with slight modification. Plastid-enriched RNA was extracted with Trizol reagent (Omic Bio, Taiwan). To eliminate DNA contamination, RNA samples were treated with DNase by use of the RapidOut DNA removal kit (Thermo, USA). The RNA quality was checked by PCR and RT-PCR before RNA-sequencing (RNA-Seq).

### NGS

Plastid-enriched RNA from leaf and floral tissues underwent NGS with Ion Proton and Illumina Hiseq 2000 platforms, respectively (Yourgene Bioscience, Taiwan). In brief, ribosomal RNA was depleted from total RNA by using RiboMinus™ eukaryote kit (Life technologies, USA), and subsequently RNA was fragmented using RNase III. The fragmented RNA was then hybridized and ligated with adaptor mixture. Subsequently, the reverse transcription was performed. The cDNA was amplified using Platinum PCR SuperMix High Fidelity reaction mix, and then the library was sequenced. In total, two independent libraries from leaf and one from floral tissue were constructed and sequenced (Additional file 1: Table S1). Short-sequenced reads were trimmed with a minimal of 35-bp read and error probability <0.05. Approximately 49,722,976 reads with average 114.9 bp and 29,726,302 reads with average 111.7 bp were obtained from two independent libraries of leaf tissues, respectively, and 21,207,630 reads with average 95.8 bp in length were obtained from floral tissues (Additional file 1: Table S1). The raw sequence reads data were submitted to the Sequence Read Archive (http://trace.ncbi.nlm.nih.gov/Traces/sra/) with BioSample Accession Numbers SRR4996537, SRR4098109 and SRR4098702.

### Determination of RNA editing sites and gene expression profile

Transcriptome analysis involved use of CLC Genomic Workbench 7.5.1 (CLC Bio, Aarhus, Denmark) as described with modification (Suzuki et al. 2013). In brief, quality control was adjusted with the mapping parameters of length fraction 0.98 and similarity 0.98 to eliminate undesirable fragment reads. The sequence reads were mapped to the cpDNA (Accession AY916449) template of *P. aphrodite* (Chang et al. 2006). The low frequency variant detection parameters were set to 5% for required significance, and the minimum coverage, minimum count and minimum frequency was set to 10, 2, and 5% respectively. Then, the position of RNA editing sites, total read counts and coverage depth were identified. The level of nucleotide conversion for each site was calculated according to the number of reads with nucleotide conversion divided by number of total reads, and frequency >5% of C-to-U or U-to-C conversions at specific nucleotide positions was defined as an RNA editing site as described in Wang et al. (2015). In addition, we manually inspect for the edits located in the regions of homopolymer runs of more than five to eliminate potentially cause by sequencing errors. Reads per kilobase of exon model per million mapped reads (RPKM) values was measured to estimate the gene expression profile as described (Mortazavi et al. 2008). To compare and validate the obtained RNA editing sites from flower tissue, we alternatively conducted a bioinformatic analysis using RES-scanner (Wang et al. 2016). In brief, the quality of RNA-seq reads from flower tissue was checked, and then trimmed using Trimmomatic (Bolger et al. 2014) version 0.33 with the following parameters: CROP:90 HEADCROP:10 LEADING:30 TRAILING:30 SLIDINGWINDOW:4:20 MINLEN:50. Subsequently, the RNA-seq reads were mapped to the reference cpDNA using RES-scanner (Wang et al. 2016) with the parameters of Phred-scaled base quality score cutoff 30 and mapping quality score cutoff 39. The RNA editing sites were filtered, and then defined the same as described above.

### Prediction of RNA structure

The tRNA structure was predicted by tRNA-scanning (Schattner et al. 2005). The secondary structure of non-coding transcripts of 100 nt in length with 50 nt extending from both upstream and downstream of indicated editing sites were predicted by using CLC Genomic Workbench 7.5.1.

## Results

### Plastid RNA edits in moth orchid

Previously, 44 RNA edits were identified from plastid protein-coding transcripts of leaf tissues in *P. aphrodite* subsp. *formosana* via RT-PCR and then conventional Sanger sequencing (Zeng et al. 2007). To sensitively and extensively examine the plastid RNA editing status of moth orchid, RNA-Seq was used to investigate the editing status of whole-plastid transcripts from leaf and floral tissues by mapping the sequence reads to the corresponding cpDNA in *P. aphrodite* subsp. *formosana*. Two independent libraries from leaf and one from floral tissue were sequenced (Additional file 1: Table S1). We mapped 805,063 (leaf-1), 1,751,673 reads (leaf-2) and 7,177,146 reads (flower) to the chloroplast DNA, which account for 1.5, 5.9 and 33% of total reads, respectively. The average genomic coverage is 591 (leaf-1), 1174 (leaf-2) and 4645 (flower) folds, respectively (Additional file 1: Table S1). The NGS libraries with genome coverage more than 1000 folds were further used for RNA editing analysis. With the threshold of at least 5% C-to-U or U-to-C conversion events observed in sequence reads considered as RNA editing sites, 137 RNA edits with 126 C-to-U and 11 U-to-C conversions, which include 93 newly discovered edits, were identified in plastid transcripts, representing an average of 0.09% of the nucleotides examined in moth orchid (Table 1; Additional file 1: Table S2). Overall, 110 and 106 edits were present in leaf and floral tissue, respectively; 79 edits, including 44 previously identified edits (Zeng et al. 2007), were commonly present in both leaf and floral tissues, whereas 31 and 27 edits were specific to leaf or floral tissues, respectively (Table 1). According to the edited proportion of each nucleotide in sequence reads with a C-to-U or U-to-C conversion after mapping to the corresponding DNA template over the total reads in that nucleotide position, the efficiency of RNA editing for each edit was further classified into five groups. Full editing was defined as >90% efficiency, high partial editing 60–90% efficiency, medium partial editing 40–60% efficiency, low partial editing 20–40% efficiency, and poor partial editing 5–20% efficiency. Overall, 10 (9.1%) and 18 (17%) fully edited sites were present in leaf and floral tissue, respectively; 26 (23.6%) and 30 (28.3%) high partially edited sites; 15 (13.6%) and 11 (10.4%) medium partially edited sites; 14 (12.7%) and 6 (5.7%) low partially edited sites; and 45 (40.9%) and 41 (39.6%) poor partially edited sites (Additional file 1: Figure S1, Table S2).

**Table 1:** Summary of plastid RNA edits in *Phalaenopsis* orchid

	Leaf	Flower	Common^a^	Total	Reported^b^
Protein coding transcripts	67	69	57	79	43
1st codon position	9	10	7	12	4
2nd codon position	47	51	43	55	39
3rd codon position	11	8	7	12	0
Creation of start codon	2	2	1	3	1
Creation of stop codon	4	5	3	6	0
Nonsynonymous substitution	50	54	46	58	42
Synonymous substitution	11	8	7	12	1
Non-protein coding transcripts	43	37	22	58	1
tRNA	1	1	1	1	0
rRNA	0	0	0	0	0
Intron	4	6	2	8	0
Intergenic spacer (IGS)	38	30	19	49	1
Total	110	106	79	137	44

^a^Common RNA edits in both leaf and floral with editing status >5%

^b^Previously reported in Zeng et al. (2007)

### RNA editing in protein-coding transcripts

In total, 42 protein-coding transcripts were responsible for 79 edits, and 67 and 69 edits were present in leaf and floral tissues, respectively, with 57 edits in common (Table 1). In particular, the *psbN* and *petL* transcripts had the highest density of RNA editing sites in both tissues, with more than 1.5% of nucleotides having various levels of RNA editing. In contrast, *ycf1*, *rpoC2* and *psaB* transcripts had the lowest density (Additional file 1: Figure S2). However, 17 edits including two newly identified sites (*rpoB*-55; *rpoC1*-1638) from the *rpo* transcripts showed the highest number among functional gene categories, and three edits (*rpoB*-55, *rpoC1*-203, *rpoC1*-1638) were unique to moth orchids as compared with 17 other species of seed plants (Additional file 1: Tables S2 and S3). Among the 79 edits that involved codons, 12 (15%) were in the first position and 55 (70%) in the second position, which resulted in 12 (15%) synonymous substitutions and 67 (85%) nonsynonymous substitutions (Table 1 and Additional file 1: Figure S3A, B). This result is consistent with previous reports regarding genome-wide RNA editing across widely divergent taxa, showing a bias in favor of second codon-position edits (Zeng et al. 2007). The most frequently edited codon was Ser converted to Leu (28%), followed by Ser to Phe (18%), and Pro to Leu (15%) resulting from nonsynonymous substitution (Additional file 1: Figure S3C). In addition, the consequence of amino acid substitution results in the increase of hydrophobicity (Additional file 1: Figure S3D).

**Fig. 1:**
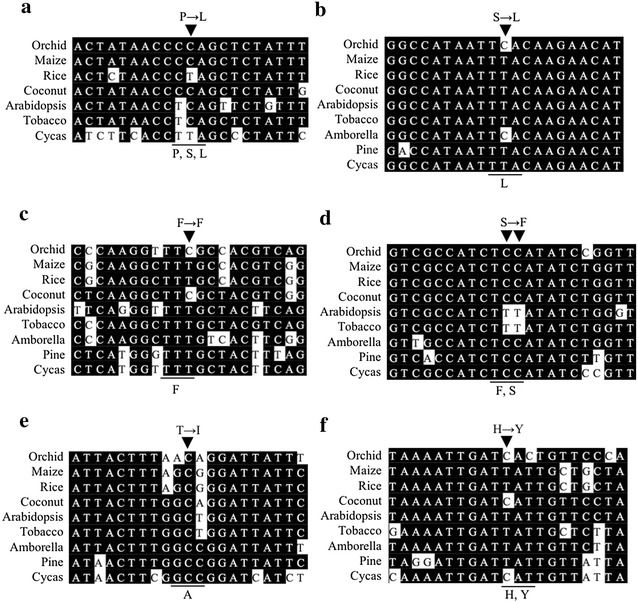
Seven newly identified edits with high editing efficiency in protein-coding transcripts of moth orchid. The upstream and downstream 10 nucleotide from the indicated edits (C to U conversion) in protein-coding transcripts of moth orchid and the corresponding regions from other eight species of seed plants were multiple aligned by using LaserGene (DNAstar, USA). **a** *petL*-56; **b** *rps12*-221; **c** *ccsA*-336; **d** *psbN*-29 and -30; **e** *petG*-56; **f** *rps3*-583. Orchid, *Phalaenopsis aphrodite*; Maize, *Zea mays*; Rice, *Oryza sativa*; Coconut, *Cocos nucifera*; Arabidopsis, *Arabidopsis thaliana*; Tobacco, *Nicotiana tabacum*; Amborella, *Amborella trichopoda*; Pine, *Pinus thunbergi*, Cycas, *Cycas taitungensis*. Note, no *petL* gene of Amborella and Pine was annotated in NCBI database. The arrowhead indicates the editing sites in orchid, and the substitution of amino acid is shown above. The underline indicates the codon position, which code for amino acids as shown underneath among plants except orchid

Seven edits in six protein coding transcripts having relatively high editing efficiency (>40%) were previous unidentified in moth orchid (Additional file 1: Table S2). Two edits, *petL*-56 and *rps12*-221 are in high editing level (>83%) both in leaf and floral tissues. Editing of the corresponding orchid *petL*-56 causing the conversion of Ser to Leu was not reported in other eight plant species which they encode Pro or Ser in the genome except rice and cycas already coding for Leu (Fig. 1a). The orchid *rps12*-221 (S → L) edit also occurred in *Amborella* (Hein et al. 2016), and the consequence of nucleotide substitution increase the amino acid conservation among plant species (Fig. 1b). Three edits *psbN*-29, *psbN*-30 and *ccsA*-336 are in medium editing level (45–54%) in leaf, but poor (8–9%) in flower. Editing of psbN-29 and psbN-30 (S → F) in moth orchid tend to increase the amino acid conservation with tobacco and Arabidopsis, and ccsA-336 (F → F) is a silent edit (Fig. 1c, d). In contrast, petG-56 and rps3-583 are in high editing level (64–72%) in flower, but relatively low (30–35%) in leaf. Editing of petG-56 (T → I) result in the increase of amino acid hydrophobicity in moth orchid, and the corresponding site encoding Ala was not edited for other eight species of seed plant (Fig. 1e). In contrast, the editing of rps3-583 (H → Y) in moth orchid tends to increase the amino acid conservation among plants except coconut and cycas (Fig. 1f).

One medium (42%) or high partial editing (71%) was involved in the creation of the *rpl2* start codon in leaf and floral tissue, respectively. In contrast, six C-to-U conversions could result in stop codons (Table 1), but their conversion efficiency was poor (Additional file 1: Table S2). For instance, the C-to-U conversion of *ndhE*-106 and *rpl20*-40 resulted in stop codons, but the editing efficiency was poor (5–20%) in both leaf and floral tissues. In addition, *ccsA*-652 and *ycf2*-3868 showed poor partial editing (11–16%) in leaf and much less editing level (0–3%) in floral tissue. In contrast, *ndhB*-106, *rpoB*-55 and *ycf4*-163 showed poor partial editing (5–10%) for the creation of a stop codon in floral tissue, but the level was much less in leaf (1–4%).

RNA editing status in transcripts of two long conserved open reading frames, *ycf1* and *ycf2*, have not been investigated in moth orchid. We found one edit and six edits for *ycf1* and *ycf2* transcripts, respectively (Additional file 1: Table S2), but the editing efficiency was poor (5–23%) in both leaf and floral tissues. *Ndh* genes, encoding the subunits of NADH dehydrogenase complex, have various degrees of nucleotide insertion/deletion and were non-functional pseudogenes in moth orchids (Chang et al. 2006; Jheng et al. 2012). Previously, only one edit was found in the *ndhB* transcripts (1977) (Zeng et al. 2007). In this study, four more edits (*ndhB*-106*, ndhD*-528*, ndhD*-740 and *ndhE*-106) were discovered in *ndh* transcripts. The editing level of *ndhB*-1977 was medium (~57%) in both leaf and floral tissues but the editing efficiency of other four newly identified sites was poor (5–17%).

### RNA editing in non-protein coding transcripts

The plastid RNA editing status of tRNA, rRNA and non-coding transcripts was previously unexplored in moth orchid, with the exception of one edit reported in the 5′UTR of *psbH* transcripts (Zeng et al. 2007). In this study, we identified 59 edits with 22 common edits present in non-protein-coding transcripts of moth orchid; 21 and 16 edits were specific to leaf and floral tissues, respectively (Table 1; Additional file 1: Table S2). Among them, one common edit was found in tRNA, seven (four in leaf and five in floral tissue, two in common) in introns, and 51 (38 in leaf and 32 in floral tissue, 19 in common) in intergenic spacer (IGS) or untranslated regions (UTRs), but no edit was found in rRNA. Previously, chloroplast protein-coding transcripts including moth orchid showed a nearest-neighbor bias towards a U_A context immediately before and after RNA editing sites (Zeng et al. 2007). However, the condition surrounding edits in non-protein-coding transcripts have not been analyzed. In this study, we found a similar U_A context bias surrounding plastid edits in both protein-coding and non–protein-coding transcripts in moth orchid (Additional file 1: Figure S4).

**Fig. 2:**
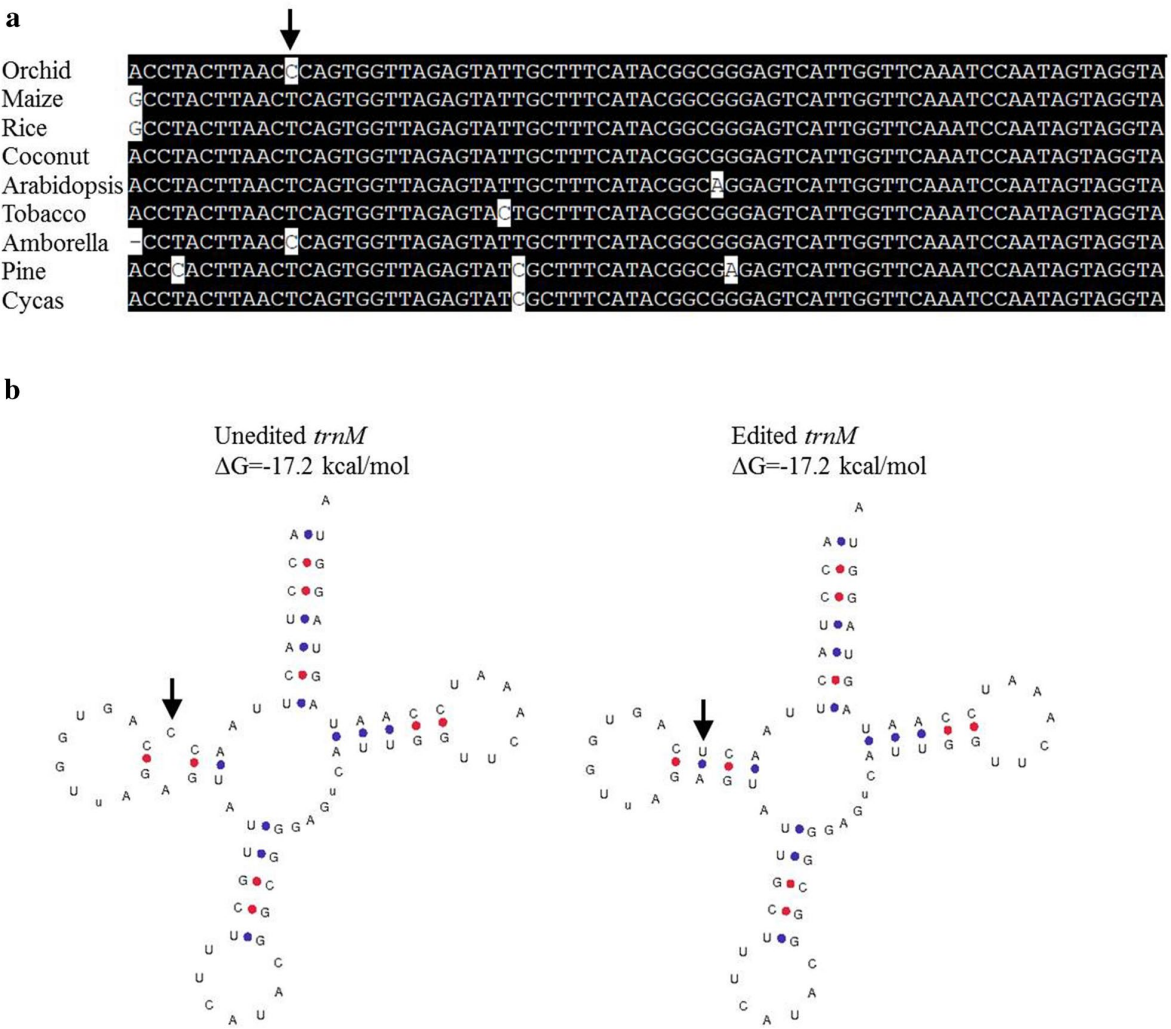
RNA editing affects the secondary structure of *trnM* transcript in moth orchid. **a** The nucleotide sequences of plastid *trnM* (cau) gene from nine species of seed plants were multiple aligned by using LaserGene (DNAstar, USA). RNA editing at the indicated position is converted from C to U in moth orchid. Orchid, *Phalaenopsis aphrodite*; Maize, *Zea mays*; Rice, *Oryza sativa*; Coconut, *Cocos nucifera*; Arabidopsis, *Arabidopsis thaliana*; Tobacco, *Nicotiana tabacum*; Amborella, *Amborella trichopoda*; Pine, *Pinus thunbergi*, Cycas, *Cycas taitungensis*. **b** The predicted tRNA secondary structures formed by the unedited and edited *trnM* transcripts, by using the tRNAscan-SE 1.21 algorithm. The edited and unedited nucleotides are indicated by arrows

Both leaf and floral tissue showed high efficiency editing (>63%) at 52,826 genomic position in *trnM* (cau) transcripts. The consequence of this C-to-U conversion in *trnM* (cau) of moth orchid resulted in increased nucleotide conservation among plant phylogeny (Fig. 2a). In addition, after being edited, despite no difference in free energy change (ΔG), the clover-leaf structure was restored as predicted by tRNAscan-SE 1.21 (Fig. 2b), which implied the functional importance of RNA editing for the *trnM*. Furthermore, we found relatively high-level editing (>48%) in the *rps12* intron (100,611) and *ycf3* intron (44,389; 45,108) (Additional file 1: Table S2). To determine whether the C-to-U conversion in these precursor transcripts affected the RNA secondary structure, the sequence extending 50 nt to both upstream and downstream of the edit in its edited and unedited form was analyzed. The secondary structures and free energy of these two transcripts, *rps12* intron (100,611) and *ycf3* intron (45,108) did not change before and after editing (Additional file 1: Figure S5A, B). In contrast, the secondary structure of *ycf3* intron (44,389) transcript was energetically more stable after editing (Additional file 1: Figure S5C).

Previously, the edit of the *psbT*-*psbH* (76,156 position) IGS, which also located in the 5′UTR of *psbH* (−30 position), resulted in the formation of an energetically less stable secondary structure (Zeng et al. 2007). We found very high efficiency editing (>82%) for this *psbH* 5′UTR edit in both leaf and floral tissues. In addition, at least a 50% editing level was found in the following seven IGS edits *clpP*-*rpl20* (70,142), *psaI*-*ycf4* (60,764), *psbB*-*psbT* (75,495), *psbM*-*rpoB* (27,736), *rps16*-*matK* (3095), *trnD*-*psbM* (31,129), and *trnN*-*rps12* (130,345) (Additional file 1: Table S2), which most of them might locate in the 5′ or 3′ UTRs and play an important role in regulation of gene expression. To determine whether the nucleotide substitution in these transcripts affected the RNA secondary structure, the sequence extending 50 nt to each side of the edit was predicted in its edited and unedited form. The secondary structure was energetically less stable in edited than non-edited transcripts of *psaI*-*ycf4* (60,764) and *rps16*-*matK* (3095). In contrast, the secondary structure was energetically more stable in edited than non-edited forms of *clpP*-*rpl20* (70,142), *psbM*-*rpoB* (27,736) as well as *trnD*-*psbM* (31,129) transcripts, but with no significant difference in edited and non-edited forms of the *psbB*-*psbT* (75,495) and *trnN*-*rps12* (130,345) transcripts were observed (Additional file 1: Figure S5D–J).

### Differential status of RNA editing

**Fig. 3:**
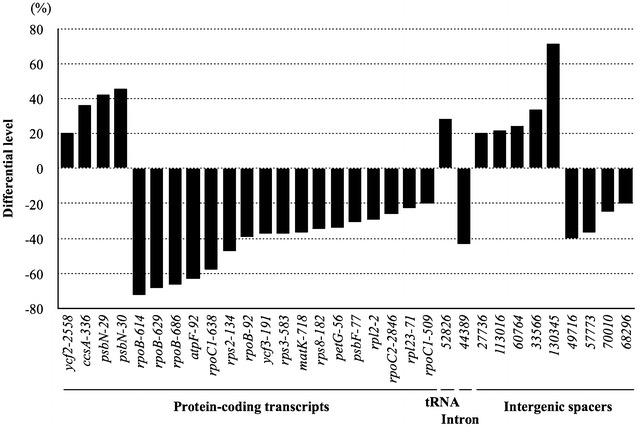
Differential status of RNA editing between leaf and floral tissues. The differential editing level (>20%) for each edit between leaf and floral tissues is shown. The bars above the x-axis indicate upregulated editing level, and those below indicate downregulated editing level in leaf tissues as compared to floral tissues

Among 137 edits, we found significantly differential efficiency (>20%) of RNA editing for at least 32 edits between leaf and floral tissue, with 10 edits upregulated and 22 edits downregulated in comparing leaf and floral tissues (Fig. 3; Additional file 1: Table S2). Among 79 edits in protein-coding transcripts, 4 edits were significantly upregulated from 20 to 46%, and 17 edits were significantly downregulated, from 20 to 72% in comparing leaf and floral tissues (Fig. 3; Additional file 1: Table S2). In particular, two edits (*psbN*-29, 30) in *psbN* transcripts were upregulated to more than 42%, and the *ccsA*-336 edit was upregulated by 36%. In contrast, *rpoB*-614, *rpoB*-629, *rpoB*-686, *atpF*-92 and *rpoC1*-638 were downregulated by 72, 68, 66, 63 and 58%, respectively, in comparing leaf and floral tissues. These results suggested the involvements of unidentified tissue-specific factors in editing of protein-coding transcripts in moth orchid.

The *trnM* (52,826) is the only edit occurred in tRNA transcripts, which it is high partial editing (64%) in floral tissue and was upregulated 29% to nearly fully edited in leaf tissue (Fig. 3; Additional file 1: Table S2). Among 7 edits in introns, one edit at *ycf3* intron (44,389) showed significant differential editing up to 43% between the two tissue types (Fig. 3; Additional file 1: Table S2). Among 51 edits in IGS, 9 differential (>20%) edits were identified: 5 edits were significantly upregulated from 20 to 71%, and 4 edits were significantly downregulated from 20 to 40% in comparing leaf and floral tissues (Fig. 3; Additional file 1: Table S2). For example, the efficiency of C-to-U conversion in the IGSs of *trnT*-*psbD* (33,566) and *trnN*-*rps12* (130,345) was upregulated to 33, and 71%, respectively, in comparing leaf and floral tissues, with the editing barely detectable in floral tissue. In contrast, the C-to-U conversion efficiency in the IGSs of *ndhJ*-*trnT* (49,716) and *rbcL*-*accD* (57,773) was downregulated by 40 and 37%, respectively, in comparing leaf and floral tissues. Meanwhile, the former edit were specific for floral tissue. Those analyses suggested the involvements of unknown tissue-specific factors in editing of none-protein-coding transcripts in moth orchid.

### Expression profile of plastid protein-coding genes in moth orchids

**Fig. 4:**
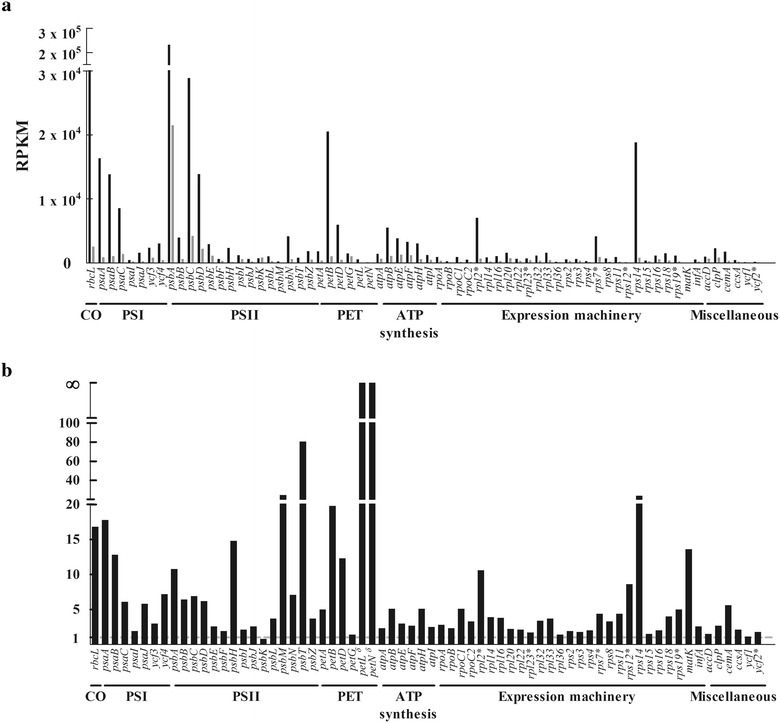
Plastid gene expression profiles in leaf and floral tissues of moth orchid by RNA-Seq. **a** The RNA-Seq sequenced reads from leaf and floral tissues were imported into CLC Genomics Workbench and mapped to the plastid 68 protein-coding genes. The measurement of leaf (black bar) and floral (grey bar) RPKM is described in “Methods”. **b** The relative expression of plastid protein-coding genes at the tissue level is shown by dividing the RPKM of leaf tissue to that of floral tissue. The asterisk indicates the genes present in the inverted repeat region of plastid genome. The δ indicate the RPKM of *petL* and *petN* is 0 in floral tissue

RNA-Seq was used to investigate the relative steady-state expression of 68 plastid protein-coding genes from leaf and floral tissues of moth orchid by RPKM analysis. In both leaf and floral tissues, photosynthesis-related genes were most highly expressed. For instance, four genes (*psbA*, *rbcL, psaC* and *petB*) were the most highly expressed, with RPKM > 20,000 in leaf and four genes (*psbA*, *psaC, rbcL* and *psbD*) were the most highly expressed, with RPKM > 2250 in flower (Fig. 4a). The expression of *psbA* was highest in both leaf and floral tissues (Fig. 4a). In contrast, the expression of *matK* was the lowest, with RPKM < 2 in leaf and the expression of *petL* and *petN* is extremely low in flower, since the RPKM is barely detectable. Most plastid genes expressed in higher level in leaf than that in flower with the exception of *psbK*. The differential level of gene expression between leaf and floral tissues could range from 0.8-fold (*psbK)* to 80-fold (*psbT*) (Fig. 4b). According to functional categories, the plastid protein-coding genes were classified into seven groups, including CO_2_ carboxylation (CO), photosystem I (PSI), photosystem II (PSII), cytochrome b_6/f_ complex (PET), ATP synthase, gene expression machinery, and miscellaneous (Fig. 4). The *rbcL* and genes coding for subunits of electron transport chain complexes had significantly higher (10.6 folds in average) expression level in leaf than floral tissue, although genes involved in other groups such as expression apparatus, ATP synthase and miscellaneous function also had higher (4.6, 3.4 and 2.5 folds, respectively) expression level in leaf than flower (Fig. 4b).

## Discussion

Previously, 44 edits were identified from 24 plastid protein-coding transcripts in leaves of *P. aphrodite* subsp. *formosana* by conventional Sanger sequencing (Zeng et al. 2007). In addition, bioinformatic analysis revealed high conservation (43 of 44) of plastid RNA editing sites between *P. equestris* and *P. aphrodite* (Jheng et al. 2012). Recently, RNA-Seq approaches were found to be more rapid, sensitive, extensive and cost-effective for investigating organellar RNA editing events than previously developed methods (Bentolila et al. 2013; Wang et al. 2015). For example, with NGS, 13, 133 and 119 new mitochondrial edits were identified from floral and leaf tissue of Arabidopsis and tobacco leaves, respectively (Bentolila et al. 2013; Grimes et al. 2014; Picardi et al. 2010). As well, up to nine new plastid edits were discovered in Arabidopsis (Bentolila et al. 2013), and many more partial edits could be detected in mitochondria of cotton than with the traditional approach (Suzuki et al. 2013). In this study, we investigated the whole plastid RNA editing status from leaf and floral tissues of *P. aphrodite* by RNA-Seq approaches. We identified 137 plastid RNA edits, including 93 newly discovered edits, which represented an average of 0.09% of the nucleotides examined in the plastid genome (Table 1; Additional file 1: Table S2). Furthermore, comparative cpDNA analysis potentially revealed high conservation (128 of 137) of plastid RNA edits between two endemic moth orchids, *P. aphrodite* and *P. equestris* (Additional file 1: Table S2). In addition, we also conducted a comparative analysis for those edits from flower tissue between two different approaches, CLC Genomics Workbench and RES-scanner (Additional file 1: Table S5). The results showed that both approaches can simultaneously identify the same 84 edits which most edits have similar editing ratio. Five or nine edits with the most of editing ratio <8.7% are specifically identified by either CLC Genomics Workbench or RES-scanner, respectively, which it is probably due to the difference for the parameters used and the algorithms of software.

Plant mitochondrial RNA editing sites are abundant and highly variable, with approximately 189–635 edits in angiosperms (Bentolila et al. 2013; Grimes et al. 2014; Sloan et al. 2010), whereas plastid RNA edits are relatively limited, with 21–44 nucleotide conversions in seed plants (Chen et al. 2011; Lin et al. 2015a, b; Sasaki et al. 2003, 2006; Tsudzuki et al. 2001; Zeng et al. 2007). All plastid edits in seed plants are the C-to-U conversion type with the exception of the oil palm *Elaeis guineensis* Jacq. (Uthaipaisanwong et al. 2012) and Antarctic hairgrass *Deschampsia antarctica* Desv. (Lee et al. 2014). However, recent reports have revealed more plastid edits in some taxa of seed plants (Additional file 1: Table S3). For example, in the gymnosperm, *Cycas taitungensis* (Chen et al. 2011) and *Ginkgo biloba* (He et al. 2016), 85 and 255 edits were identified from protein-coding transcripts, respectively. In dicot, cotton *Gossypium hirsutum*, 54 edits were identified in 27 transcripts (Jiang et al. 2012). In monocot, coconut *Cocos nucifera* L., duckweed *Spirodela polyrhiza* and Tausch’s goatgrass *Aegilops tauschii L.*, 75, 66 and 60 plastid RNA edits were identified, respectively (Huang et al. 2013; Wang et al. 2015, 2017). In the early-branching angiosperm *Amborella trichopoda,* 138 edits were revealed (Hein et al. 2016). Here we identified 137 edits with 126 C-to-U and 11 U-to-C conversion from moth orchid, the highest number so far reported in monocot (Table 1), though nearly 57% of edits showed poor-level (5–20%) editing (Additional file 1: Figure S1, Table S2). However, chloroplast RNA editing is highly variable, with more edits in lower taxa of land plants. For instance, in hornwort *Anthoceros formosae*, at least 943 had 509 (54%) C-to-U and 433 (46%) U-to-C conversions (Kugita et al. 2003). Lycophyte showed a large number of plastid RNA editing sites, up to 3415 edits with only C-to-U conversion in the spike moss *Selaginella uncinata* (Oldenkott et al. 2014). In the fern *Adiantum capillus*-veneris, 350 RNA editing sites with 90% C-to-U edits and 10% U-to-C edits were identified (Wolf et al. 2004). Additionally, the plastid transcriptomes analysis from two early diverging species of fern, *Ophioglossum californicum* and *Psilotum nudum,* identified 297 C-to-U and three U-to-C edits in the former plastid transcripts, but only 27 C-to-U and no U-to-C edits in the latter plastid transcripts (Guo et al. 2015). These analyses suggest an independent gain or loss of RNA edits across the taxa of land plants during evolution.

RNA editing occurs most frequently in the *ndh* transcripts, accounting for >40% of the edits in the chloroplast transcripts of most higher plants with the exception of *Pinus thunbergii* and *Phalaenopsis aphrodite* (Chen et al. 2011; Huang et al. 2013; Lin et al. 2015a, b; Wakasugi et al. 1996; Wang et al. 2015; Zeng et al. 2007) because the functional *ndh* genes were lost from the plastids of these two species (Chang et al. 2006; Wakasugi et al. 1994). In Arabidopsis, those *ndh* edits are differentially regulated in distinct tissue types (Tseng et al. 2013). In this study, only a small fraction (5 of 137) of RNA edits were contributed from *ndh* transcripts in moth orchid, and they also showed poor editing efficiency (3–14%), with the exception of the previously identified *ndhB*-1977 edit (Additional file 1: Table S2). In addition, two sites (*ndhD*-528 and -740) in *ndhD* transcript were completely undetectable in floral tissue (Additional file 1: Table S2), which suggested that some tissue-specific factors may regulate the editing process of *ndh* transcripts in moth orchid. Additionally, the remaining five partial edits in *ndh* transcripts are likely an evolutionary remnant from before the complete loss of plastid RNA edits for non-functional *ndh* pseudogenes in moth orchid.

Previous study showed that the *rpo* (*rpoA*, *rpoB*, *rpoC1* and *rpoC2)* transcripts were the most extensively edited (15 sites, up to 34%) among the functional gene groups in moth orchid (Zeng et al. 2007). We discovered two new edits (*rpoB*-55; *rpoC1*-1638) from *rpo* transcripts, although their editing level was poor (~10%) (Additional file 1: Tables S2, S4). In addition, five edits (*rpoB*-55, -*614,* -*629,* -*686* and *rpoC1*-1638) were preferentially occurred in floral tissues. Furthermore, three edits (*rpoB*-55, *rpoC1*-203 and *rpoC1*-1638) were unique to moth orchid as compared with 17 other seed plants (Additional file 1: Table S4), although their editing level was poor with the exception of *rpoC1*-203. In contrast, the four nucleotides (*rpoA*-527, *rpoB*-1241, *rpoC1*-243, -656) of *rpo* transcripts were edited or T is present in the corresponding DNA in most of seed plants but are not edited in moth orchid, which caused the divergence of amino acid residues between moth orchid and other seed plants (Additional file 1: Table S4), though the significance is unknown.

Previous study has demonstrated that the pattern and efficiency of some editing sites in organellar transcripts significantly differed among ecotypes, tissue types, developmental stages and environmental conditions (Bentolila et al. 2005; Chateigner-Boutin and Hanson 2003; Howad and Kempken 1997; Ruf and Kossel 1997; Tillich et al. 2005; Tseng et al. 2013). For instance, in Arabidopsis, the editing of *ndhB*-149, *ndhB*-1255, and *ndhD*-2 occurs in leaf but is completely lost in roots and in lincomycin-treated seedlings (Tseng et al. 2013). In this study, at least 32 sites showed significantly differential (≧20%) RNA editing between leaf and floral tissues in moth orchid (Fig. 3; Additional file 1: Table S2). In addition, 19 and 14 edits are unique for leaf and floral tissues, respectively, which RNA editing is completely undetectable in floral tissue or vice versa (Additional file 1: Table S2). This analysis suggested tissue-specific factors might involve in RNA editing process in moth orchid.

Previously, a C-to-U conversion at the −10 position of the *ndhG* 5′UTR in monocot plants was predicted to modify the RNA secondary (stem/loop) structure (Drescher et al. 2002), but was not observed in moth orchid. Instead, a C-to-U substitution at the −30 position of the *psbH* 5′UTR that can form an energetically less stable secondary structure was reported in moth orchid (Zeng et al. 2007). Therefore, edits that occurred in the UTR regions might play an important role in the regulation of gene expression. We found very high efficiency editing (>81%) at the *psbH* 5′UTR (−30) in both leaf and floral tissues (Additional file 1: Table S2). In addition, at least 50% editing efficiency was observed for the edits of IGSs such as *psaI*-*ycf4* (60,764), *psbM*-*rpoB* (27,736), *trnD*-*psbM* (31,129), *rps16*-*matK* (3095), *clpP*-*rpl20 (70,142)* and *psbB*-*psbT* (75,495) located in the 3′ or 5′UTR of genes such as *psaI* (27, 27 nt downstream of stop codon), *rpoB* (−160, 160 nt upstream of start codon), *psbM* (−85), *matK* (−35), *rpl20* (−134), *psbB* (29), respectively. In comparing the structure of edited and unedited transcripts, significant change in free energy was predicted for the former five (Additional file 1: Figure S5D–H), which suggested that some edits occurring at the 3′ or 5′UTR might play a role in the regulation of gene expression in moth orchid.

Previous study reported that some sites of intron editing are a prerequisite for RNA splicing in plant organelles (Borner et al. 1995; Farre et al. 2012; Vogel et al. 1997), and unspliced RNA is often only partially edited (Yang and Mulligan 1991). In this study, 8 RNA edits were identified in seven introns (Additional file 1: Table S2), in particular, edits in *rps12* (100,611) and *ycf3* (45,108) introns showed high level (>73%) of editing. In addition, leaf-specific editing was found in the *clpP* intron (71,815) and flower-specific editing in the *clpP* intron (72,384), though the editing level is poor. Furthermore, the edit *ycf3* (44,389) in intron showed significantly (>43%) differential editing between leaf and floral tissues (Fig. 3; Additional file 1: Table S2). These analyses suggested that editing in some *cis*-elements of primary transcripts might be important for splicing, and unidentified tissue-specific factors might be required for regulating intron editing in moth orchid. Additionally, RNA editing occurs in tRNA in plant organelles and plays an important role in tRNA processing and maturation (Binder et al. 1994; Kunzmann et al. 1998; Marechal-Drouard et al. 1996). The editing of the tRNA precursor might occur in the anticodon stem, acceptor stem, D stem or even anticodon (Janke and Paabo 1993; Marechal-Drouard et al. 1996). In this study, we observed nearly full RNA editing at the D stem of *trnM* transcripts (52,826 position) in leaf, with significant (>29%) differential editing between leaf and floral tissues (Fig. 3; Additional file 1: Table S2), which suggested that tissue-specific tRNA editing factors might be involved. After being edited in moth orchid, the *trnM* sequence conservation among plant phylogeny was increased, with the exception of *Amborella* species, then the standard clover-leaf tRNA structure could be formed (Fig. 2). The corresponding edit might also occur in *Amborella*, although this has not been reported (Hein et al. 2016).

The Arabidopsis editosome consists of at least four nucleus-encoded protein families, PPR, RIPs, ORRM1 and OZ1, required for plant organelle RNA editing (Sun et al. 2015, 2016). The PPR protein family with more than 400 members in angiosperms is involved in RNA metabolism including RNA editing in plant organelles (Grennan 2011). Although no PPR proteins involved in RNA metabolism have been reported in orchids, approximately 254 expressed sequence tags (EST) potentially encoding PPR proteins in moth orchid were annotated in the Orchidbase database (http://orchidbase.itps.ncku.edu.tw/est/home2012.aspx). In addition, at least three RIP homologues, five OZ1 homologues and two ORRM1-like proteins are available in Orchidbase. Our analysis suggests that the editosome of moth orchids might resemble that of Arabidopsis.

Chloroplast transcription change in large-scale in response to environmental or developmental signals (Barkan 2011). In Arabidopsis, the steady state levels of most plastid gene expression were high in green tissues, while low or undetectable in non-green tissues (Tseng et al. 2013). However, appreciable levels of some plastid transcripts such as *clpP* and *rps14* mRNAs could be detected in non-photosynthetic tissue (Tseng et al. 2013). In this study, the differential steady state level of plastid transcripts between leaf and floral tissues could range from 0.8 to 80 fold (Fig. 4b), with most plastid genes having higher expression level in leaf than floral tissue, with the exception of *psbK*. In addition, genes located in the inverted repeat regions did not show higher expression level than those in single copy regions (Fig. 4). Chloroplast gene expression is regulated at multiple steps such as transcription, splicing, editing, processing, stability, translation, and post-translational modification, and no single step is the primary regulated step (Barkan 2011). Rather, each step might contribute to distinct patterns of plastid gene expression under different environmental and developmental conditions. Therefore, the variation of gene copy number and steady state transcript level probably did not reflect to the protein level in the plastids of distinctive tissues in moth orchid. Further studies are required to clarify how plastid gene expression is regulated in moth orchid.

## Conclusions

We have identified 137 edits including 93 newly discovered edits in plastid transcripts of moth orchid by RNA-Seq. It is the highest number reported so far in monocots. Overall, 79 edits were involved in protein-coding transcripts, and the consequence of 58 nucleotide conversions caused the non-synonymous substitution which tend to increase the amino acid hydrophobicity as well as conservation among plant phylogeny. RNA editing occurred in non-protein-coding transcripts such as tRNA, introns and untranslated regulatory regions could affect the formation and stability of secondary structure, which might play a regulatory role in gene expression. For instance, RNA editing in *trnM* is required for the formation of a standard clover-leaf structure. The nearest-neighbor bias towards a U_A context immediately before and after RNA edits occurred in both protein-coding and non-protein-coding transcripts in moth orchid. At least 32 edits showed significant (≧20%) differential editing between leaf and floral tissues, which suggested that some unidentified tissue-specific factors might be responsible for the regulation of RNA editing in moth orchid. Furthermore, most plastid genes expressed in higher level in leaf than that in flower, but it might not reflect to the protein level in the plastids of distinctive tissues in moth orchid.

## Additional file

**Additional file 1: Table S1.** Statistics of NGS libraries mapped to reference genome. **Table S2.** Plastid RNA edits in *Phalaenopsis* orchid. **Table S3.** Plastid RNA edits in protein-coding transcripts among land plants. **Table S4.** Plastid RNA edits in *rpo* transcripts among 18 species of higher plants. **Table S5.** Comparative analysis of plastid RNA edits from flower tissue by two different bioinformatic approaches. **Figure S1.** The editing efficiency of plastid RNA edits in leaf and floral tissues. **Figure S2.** The density of RNA editing sites in protein-coding transcripts. **Figure S3.** RNA editing in protein-coding transcripts. **Figure S4.** Nearest-neighbor bias toward a U_A context immediately before and after plastid RNA edits in moth orchid. **Figure S5.** The prediction of RNA secondary structures formed by the unedited and edited plastid transcripts in moth orchid.

## Abbreviations

cpDNA: chloroplast DNA
DOC: depth of coverage
IGS: intergenic spacer
mtDNA: mitochondrial DNA
MORF: multiple organellar RNA editing factor
NGS: next generation sequencing
PPR: pentatricopeptide repeat protein
RHS: Royal Horticultural Society
RIP: RNA editing-interacting protein
RNA-Seq: RNA sequencing
UTR: untranslated regulatory region
