# Supplementary material for: Whole plastid transcriptomes reveal abundant RNA editing sites and differential editing status in Phalaenopsis aphrodite subsp. formosana
Source: Bot Stud. 2017 Sep 16;58:38. doi: 10.1186/s40529-017-0193-7 (PMC5602750; doi:10.1186/s40529-017-0193-7)
Supplement: Supplementary file 1 — Additional file 1: Table S1. Statistics of NGS libraries mapped to reference genome. Table S2. Plastid RNA edits in Phalaenopsis orchid. Table S3. Plastid RNA edits in protein-coding transcripts among land plants. Table S4. Plastid RNA edits in rpo transcripts among 18 species of higher plants. Table S5. Comparative analysis of plastid RNA edits from flower tissue by two different bioinformatic approaches. Figure S1. The editing efficiency of plastid RNA edits in leaf and floral tissues. Figure S2. The density of RNA editing sites in protein-coding transcripts. Figure S3. RNA editing in protein-coding transcripts. Figure S4. Nearest-neighbor bias toward a U_A context immediately before and after plastid RNA edits in moth orchid. Figure S5. The prediction of RNA secondary structures formed by the unedited and edited plastid transcripts in moth orchid. [file 40529_2017_193_MOESM1_ESM.pdf]

Supplementary Table 1. Statistics of NGS libraries mapped to reference genome (Accession: AY916449)

Library	SRA number	NGS methods	Total reads	Total number of bases	Mapped reads					
					Counts	Percentage of reads	Average length	Number of bases	Percentage of bases	Coverage
**Leaf-1**	SRR4996537	Ion proton	49,722,976	5,715,271,000	805,063	1.62%	109.39	88,065,279	1.54%	591.2
**Leaf-2**	SRR4098109	Ion proton	29,726,302	3,320,042,839	1,751,673	5.89%	99.86	174,929,016	5.27%	1174.3
**Flower**	SRR4098702	Illumina	21,761,063	2,078,933,675	7,177,146	32.98%	96.40	691,885,249	33.28%	4644.6

Supplementary Table 2. Plastid RNA edits in *Phalaenopsis* orchid

Gene[ψ]	Genome position[c]	Gene position[a]	Edited codon	Edited position	Amino acid change	Leaf[d]		Flower		Difference (%)
						Edited ratio (%)	Coverage	Edited ratio (%)	Coverage	
accD	58734	504	gaC→gaU	3	D→D	15.4	39	15.7	249	-0.3
	59414	1184[a]	uCa→uUa	2	S→L	89.2	83	96.4	420	-7.3
	59642	1412[a]	cCa→cUa	2	P→L	79.4	102	96.9	418	-17.5
	59660	1430[a]	cCu→cUu	2	P→L	78.6	112	84.9	245	-6.3
atpA	11667	773[a]	uCa→uUa	2	S→L	88.7	274	94.8	465	-6.2
	11292	1148[a]	uCa→uUa	2	P→L	64.8	128	83.9	254	-19.0
atpB	54954	15	ccC→ccU	3	P→P	6.0	352	1.5	654	4.5
	53785	1184[a]	uCa→uUa	2	S→L	97.4	852	97.6	1263	-0.1
atpF	13962	92[a]	cCa→cUa	2	P→L	20.5	336	84.0	700	-63.5
atpI	15480	428[a]	cCu→cUu	2	P→L	99.0	197	99.5	413	-0.5
	15279	629[a]	uCa→uUa	2	S→L	100.0	84	97.6	423	2.4
ccsA	113255	336	uuC→uuU	3	F→F	45.0	160	9.0	155	36.0
	113571[c]	652	Caa→Uaa	1	Q→stop	10.9	55	3.0	198	7.9
clpP	72255	82[a]	Cau→Uau	1	H→Y	83.2	536	92.0	363	-8.8
	71089	559[a]	Cau→Uau	1	H→Y	94.1	341	84.5	682	9.7
matK	2528	533[a]	uCu→uUu	2	S→F	55.4	473	74.3	526	-18.9
	2343	718[a]	Cau→Uau	1	H→Y	49.4	443	86.3	700	-36.9
	1995	1066[a]	Cac→Uac	1	H→Y	91.9	160	94.1	643	-2.2
ndhB[ψ]	97389	1977[a]	acC→acU	3	T→T	56.4	39	56.8	44	-0.4
	135662	106	Caa→Uaa	1	Q→stop	2.7	74	9.9	71	-7.2
ndhD[ψ]	114790	528	cuU→cuC	3	L→L	5.4	37	0.0	266	5.4
	114578[c]	740	uCu→uUu	2	S→F	5.6	107	0.0	213	5.6
ndhE[ψ]	116438[c]	106	Caa→Uaa	1	Q→stop	13.7	139	17.3	162	-3.6
petB	77940	611[a]	cCa→cUa	2	P→L	93.8	1983	96.8	1896	-3.0
petG	67192	56	aCa→aUa	2	T→I	30.2	981	64.1	3340	-34.0
petL	66862	5[a]	cCu→cUu	2	P→L	39.6	328	46.8	1080	-7.1
	66913	56	cCa→cUa	2	P→L	83.8	394	84.7	2691	-1.0
psaB	39498	1451	cCg→cUg	2	P→L	3.7	1307	7.2	725	-3.5
psaI	60705	80[a]	uCu→uUu	2	S→F	72.3	94	92.3	761	-19.9
psaJ	68176	113	uCa→uUa	2	S→L	12.6	151	8.3	588	4.3
psbC	36287	852	uuC→uuU	3	F→F	3.4	4787	13.4	2501	-10.0
psbE	65604	57	uaC→uaU	3	Y→Y	5.3	682	7.1	1163	-1.8
psbF	65322	77[a]	uCu→uUu	2	S→F	52.8	318	83.3	1101	-30.5
psbJ	64925	102	ucC→ucU	3	S→S	28.0	346	17.4	1207	10.6
psbN	76049	29	uCc→uUc	2	S→F	50.4	712	7.9	858	42.5
	76048	30	ucC→ucU	3	S→S	53.6	713	8.1	853	45.5
rpoA	80516	200[a]	uCu→uUu	2	S→F	82.1	78	78.9	199	3.2
	80348	368[a]	uCa→uUa	2	S→L	84.8	92	85.4	301	-0.6
	79886	830[a]	uCa→uUa	2	S→L	70.0	20	76.2	294	-6.2
rpoB	27521	55	Cag→Uag	1	Q→stop	4.3	70	10.4	77	-6.1
	27484	92[a]	uCc→uUc	2	S→F	16.2	37	55.4	74	-39.2
	27175	401[a]	uCu→uUu	2	S→F	84.2	19	76.4	55	7.8
	27040	536[a]	uCg→uUg	2	S→L	81.8	11	71.1	76	10.8
	26962	614[a]	uCa→uUa	2	S→L	0.0	0	72.2	36	-72.2
	26947	629[a]	uCg→uUg	2	S→L	0.0	2	68.3	41	-68.3
	26890	686[a]	cCg→cUg	2	P→L	0.0	0	66.3	80	-66.3
	25087	2489[a]	uCa→uUa	2	S→L	81.8	11	76.5	85	5.3
rpoC1	24233	62[a]	uCa→uUa	2	S→L	57.5	167	72.2	126	-14.7
	24092	203[a]	uCu→uUu	2	S→F	83.3	12	81.7	208	1.6
	23028	509[a]	cCg→cUg	2	P→L	64.5	62	84.8	145	-20.3
	22899	638[a]	uCg→uUg	2	S→L	20.5	78	78.1	128	-57.6
	21899	1638	uCa→uUa	2	S→L	0.7	140	10.3	97	-9.6
rpoC2	18443	2846[a]	uCu→uUu	2	S→F	11.8	76	37.6	93	-25.8
rps2	16782	134[a]	aCa→aUa	2	T→I	43.5	23	90.8	304	-47.3
rps3	85546	30	uuC→uuU	3	F→F	20.3	64	5.3	188	15.0
	84993	583	Cac→Uac	1	H→Y	35.0	120	72.2	115	-37.2
rps8	82172[c]	182[a]	uCa→uUa	2	S→L	53.9	39	88.8	215	-35.0
rps12	134000	221	uCa→uUa	2	S→L	85.9	391	89.7	850	-3.7
rps14	38459	149[a]	uCa→uUa	2	S→L	75.7	1909	93.5	688	-17.8
rps15	117323	17	uCa→uUa	2	S→L	0.0	33	14.2	268	-14.2
rps16	5026	143[a]	uCa→uUa	2	S→L	84.5	239	90.3	765	-5.8
rpl2	88243	2[a]	aCg→aUg	2	T→M	41.5	224	71.0	348	-29.5

rpl20	69969	40	Cga→Uga	1	R→stop	7.4	175	13.3	738	-5.9
	69674	335	uCa→uUa	2	S→L	12.9	62	9.0	456	3.9
rpl22	85813	176	uCu→uUu	2	S→F	0.0	161	6.5	294	-6.5
rpl23	146448	71[a]	uCu→uUu	2	S→F	65.8	38	88.4	258	-22.6
rpl32	112003	110	uCu→uUu	2	S→F	7.7	272	13.9	404	-6.1
ycf1	121980	1232	uCa→uUa	2	S→L	0.0	8	8.7	46	-8.7
ycf2	89754	903	ucU→ucC	3	S→S	12.1	33	0.0	59	12.1
	90914	2063	uCg→uUg	2	S→L	7.2	83	6.5	31	0.8
	91409	2558	gUu→gCu	2	V→A	23.1	13	2.5	40	20.6
	92719	3868	Cag→Uag	1	Q→stop	15.8	19	0.0	39	15.8
	92994	4143	auU→auC	3	I→I	11.1	9	0.0	31	11.1
	95393	6542	gCa→gUa	2	A→V	5.1	78	0.0	37	5.1
ycf3	45758	44[a]	uCu→uUu	2	S→F	94.4	143	93.2	749	1.2
	44886	185[a]	aCg→aUg	2	T→M	90.0	480	94.9	811	-4.9
	44880	191[a]	cCa→cUa	2	P→L	44.1	469	81.8	824	-37.7
ycf4	61295	163	Cag→Uag	1	Q→stop	1.1	441	5.1	410	-4.0
	61485	353	cUu→cCu	2	L→P	8.2	317	0.4	277	7.8
**Sub-total** [b]						67		69		
trnM(cau)	52826[e]		C→U			92.2	740	63.6	44	28.5
clpP intron1	72384		C→U			0.0	71	11.0	309	-11.0
clpP intron2	71815		C→U			5.0	120	0.0	348	5.0
rpl16 intron1	84617		C→U			2.4	126	9.8	225	-7.4
rpoC1 intron1	23369		C→U			7.3	41	1.6	64	5.7
rps12 intron1	100465		C→U			4.5	425	5.4	615	-0.9
rps12 intron1	100611[e]		C→U			73.4	492	54.4	274	19.0
ycf3 intron1	45108[e]		C→U			79.8	351	86.4	279	-6.6
ycf3 intron2	44389[e]		C→U			4.4	248	47.8	753	-43.4
accD-psaI	59761	3'UTR of *accD*	C→U			28.0	25	45.5	268	-17.5
ccsA-trnN(guu)	117201	3'UTR of *ccsA*	C→U			2.9	69	8.6	209	-5.7
ccsA-trnN(guu)	117428[e]	3'UTR of *ccsA*	C→U			13.3	15	1.6	128	11.7
ccsA-trnN(guu)	123231	5'UTR of *trnN(guu)*	C→U			0.0	0	6.9	29	-6.9
cemA-petA	62818	3'UTR of *cemA*	C→U			6.9	218	14.9	342	-8.0
clpP-rpl20	70010	5'UTR of *rpl20*	C→U			2.3	128	26.8	694	-24.5
clpP-rpl20	70142[e]	5'UTR of *rpl20* (-134 bp	C→U			93.4	1836	90.8	650	2.6
clpP-rpl20	70726	3'UTR of *clpP*	C→U			20.4	521	23.9	964	-3.5
ndhD-trnN(guu)	113016	3'UTR of *ndhD*	C→U			24.0	25	2.5	160	21.5
ndhJ-trnT(ugu)	49716	3'UTR of *ndhJ*	C→U			0.0	19	40.0	10	-40.0
petD-trnH(gug)	85997	5'UTR of *trnH(gug)*	U→C			0.4	238	12.6	454	-12.2
petL-petG	67109	5'UTR of *petG*	C→U			17.2	1701	9.5	926	7.7
psaI-ycf4	60764[e]	3'UTR of *psaI* (27 bp)	C→U			83.7	362	59.2	721	24.5
psaJ-rpl33	68296	3'UTR of *psaJ*	C→U			0.0	41	20.4	613	-20.4
psbB-psbT	75495[e]	3'UTR of *psbB* (29 bp)	C→U			48.5	33	58.3	24	-9.9
psbI-trnG(ucc)	8642	3'UTR of *psbI*	C→U			5.1	98	0.0	44	5.1
psbI-trnG(ucc)	9444	5'UTR of *trnG(ucc)*	C→U			5.0	40	3.5	198	1.5
psbK-psbI	7837	5'UTR of *psbI*	C→U			20.4	496	13.4	610	6.9
psbM-rpoB	27736[e]	5'UTR of *rpoB* (-160 bp)	C→U			51.0	49	30.9	81	20.2
psbN-psbH	76156[a]	5'UTR of *psbH*	C→U			81.8	841	92.7	930	-10.9
rbcL-accD	57581	3'UTR of *rbcL*	C→U			7.7	26	0.0	165	7.7
rbcL-accD	57773	3'UTR of *rbcL*	C→U			9.5	42	46.2	171	-36.7
rpl2-rps19	86586	5'UTR of *rps19*	C→U			27.0	111	22.7	300	4.4
rps16-matK	3095[e]	5'UTR of *matK* (-35 bp)	C→U			86.3	248	91.8	449	-5.5
rps19-rpl22	86046	5'UTR of *rpl22*	C→U			7.3	219	9.8	661	-2.5
rps25-psbK	6898	5'UTR of *psbK*	C→U			8.2	49	6.3	32	1.9
rps4-ycf3	46170	5'UTR of *ycf3*	C→U			11.1	108	1.3	75	9.8
rps4-ycf3	46179	5'UTR of *ycf3*	C→U			6.5	107	0.0	78	6.5
rps4-ycf3	46254	5'UTR of *ycf3*	C→U			6.5	46	0.0	63	6.5
trnC(gca)-petN	29655	5'UTR of *petN*	C→U			13.3	15	0.0	72	13.3
trnD(gac)-psbM	31129[e]	5'UTR of *psbM* (-85 bp)	C→U			51.5	173	51.3	263	0.1
trnG(gcc)-trnS(gga)	38003	3'UTR of *trnG(gga)*	C→U			0.7	139	7.7	26	-7.0
trnH(gug)-ycf2	88550	3'UTR of *trnH(gug)*	C→U			0.0	36	7.9	267	-7.9
trnH(gug)-ycf2	88588[e]	3'UTR of *trnH(gug)*	C→U			0.0	16	5.3	169	-5.3
trnN(guu)-rps12	124554	3'UTR of *trnN(guu)*	C→U			9.5	42	0.1	1155	9.5
trnN(guu)-rps12	125089		C→U			5.1	592	0.0	32616	5.1
trnN(guu)-rps12	126889		U→C			6.2	226	0.1	100609	6.1
trnN(guu)-rps12	130840		C→U			8.7	104	8.1	10309	0.6
trnN(guu)-rps12	131616		U→C			9.8	306	0.0	54559	9.8
trnN(guu)-rps12	132748		U→C			16.4	61	0.0	4	16.4
trnN(guu)-rps12	130345[e]		U→C			71.4	91	0.1	4648	71.3
trnR(acg)-rpl32	111392	5'UTR of *rpl32*	C→U			0.0	3	6.7	30	-6.7

trnR(acg)-ycf1	123299	5'UTR of *ycf1*	C→U			0.0	8	8.7	23	-8.7
trnR(acg)-ycf1	123418	5'UTR of *ycf1*	C→U			16.2	60	0.0	58	16.2
trnR(ucu)-trnC(gca)	10833	3'UTR of *trnR (ucu)*	C→U			5.8	69	0.0	10	5.8
trnR(ucu)-trnC(gca)	14539	3'UTR of *trnR(ucu)*	C→U			6.5	31	0.0	332	6.5
trnT(aca)-rps4	47504[c]	5'UTR of *rps4*	C→U			14.3	7	7.7	26	6.6
trnT(ggu)-psbD	33566[c]	5'UTR of *trnT(ggu)*	U→C			33.3	18	0.0	10	33.3
ycf2-trnV(gac)	95813[c]	3'UTR of *ycf2*	C→U			5.8	69	7.1	42	-1.3
Sub-total [b]						44		37		
**Total [b]**						111		106		

[a]Previously reported in Zeng *et al*., 2007.

[b]The number of edits with the level of C-to-U or U-to-C conversion more than 5% was counted.

[c]Comparative cpDNA analysis showed that this position is not edited in *P. equestris*.

[d]Mapped results from second NGS library

[e]Secondary structure were analyzed.

[Ψ]The indicated *ndh* genes are truncated pseuddogenes in moth orchid.

Supplementary Table 3. Plastid RNA edits in protein-coding transcripts among land plants.

		Hornwort	Lycophytes	Fern	Gymnosperm			Basal angiospe	Dicotyledon							Monocotyledon							
**Species[1-21]**		***Anthoceros formosae*[1]**	***Selaginella uncinata*[2]**	***Adiantum capillus-veneris*[3]**	***Cycas taitungensis*[4]**	***Pinus thunbergii*[5]**	***Ginkgo biloba*[6]**	***Amborella trichopoda*[7]**	***Arabidopsis thaliana*[8,9]**	***Nicotiana tabacum*[10]**	***Pisum sativum*[11]**	***Atropa belladonna*[12]**	***Solanum lycopersicum*[13]**	***Gossypium hirsutum*[14]**	***Vigna radiata*[15]**	***Zea mays*[16]**	***Oryza sativa*[17]**	***Cocos nucifera*[18]**	***Saccharum officinarum*[19]**	***Spirodela polyhiza*[20]**	***Elaeis guineensis*[21]**	***Deschampsia antarctica*[22]**	***Phalaenopsis aphrodite***
**Types of protein-coding transcripts**																							
Photosynthesis	Photosystem I (*psa*)	39	151	3	0	1	5	1	0	0	1	0	0	1	1	0	0	2	0	0	0	0	3
	Photosystem II (*psb*)	78	392	24	5	3	24	6	3	2	2	1	2	3	3	0	0	0	0	3	0	2	6
	Cyctochrome $b_{6/f}$ complex (*pet*)	63	207	19	6	9	14	7	1	1	2	1	1	3	2	1	0	2	1	1	1	2	4
	ATP synthase (*atp*)	102	332	44	0	6	12	11	1	3	1	2	1	4	2	1	1	6	1	1	2	4	7
	NADH-dehydrogenase complex (*ndh*)	278	795	79	14	0	96	49	18	19	13	19	20	23	18	12	13	31	12	31	5	7	5
Transcription and translation	RNA polymerase (*rpo*)	18	496	49	4	0	27	26	5	7	4	8	8	5	7	5	3	14	4	6	0	5	17
	Ribosomal small subunits (*rps*)	64	205	27	2	1	24	13	3	4	3	4	4	6	4	2	2	7	2	6	6	5	8
	Ribosomal large subunits (*rpl*)	38	102	15	0	0	10	7	1	1	0	0	0	3	1	2	1	2	2	5	4	2	6
	Translation initiation factor (*inf*)	3	0	3	0	0	1	0	0	0	0	0	0	0	0	0	0	0	0	0	0	0	0
Others	Chlorophyll biosynthesis (*chl*)	70	269	26	0	0	16	0	0	0	0	0	0	0	0	0	0	0	0	0	0	0	0
	Acetyl-CoA carboxylase (*acc*)	27	0	16	2	0	5	6	2	0	1	0	0	2	1	0	0	2	0	0	0	0	4
	Maturase (*mat*)	0	47	5	1	0	8	0	1	0	0	0	0	3	0	0	0	2	0	0	0	1	3
	Chloroplast envelope membrane protein (*cem*)	0	0	0	0	0	3	0	0	0	0	0	0	0	0	0	0	0	0	0	0	0	0
	ATP-dependent protease proteolytic subunit (*clp*)	14	37	7	2	0	5	2	1	0	0	0	0	1	1	0	0	2	0	0	2	0	2
	Cytochrome c biogenesis protein (*ccs*)	9	92	12	0	0	5	4	0	0	0	0	0	0	0	0	0	0	0	0	0	0	2
	RuBisCO large subunit (*rbcL*)	0	54	0	0	0	0	0	0	0	0	0	0	0	0	0	0	0	0	0	0	0	0
	Conserved ORFs (*ycf*)	51	231	21	1	3	20	6	1	0	0	0	0	0	0	2	1	5	1	5	8	1	12
**Total edits**		942	3415	349	37	26	255	138	37	37	27	35	36	54	40	26	21	75	23	58	28	29	79
C to U (%)		54.0	100	90.0	100	100	100	100	100	100	100	100	100	100	100	100	100	100	100	100	75.0	58.6	93.7
1st codon edits (%)		38.4	29.7	26.0	0.0	26.9	24.7	18.1	16.2	5.4	7.4	2.9	5.6	11.1	12.5	4.0	4.8	16.0	4.3	10.3	14.3	24.1	15.2
2nd codon edits (%)		58.6	60.0	68.0	100.0	73.1	69.0	78.3	78.4	91.9	92.6	97.1	94.4	87.0	85.0	92.0	95.2	82.7	95.7	84.5	50.0	48.3	69.6
3rd codon edits (%)		3.0	10.3	6.0	0.0	0	6.3	3.6	5.4	2.7	0	0	0	1.9	2.5	4.0	0.0	1.3	0	5.2	35.7	27.6	15.2
Silent edits		28	352	21	0	0	16	6	0	0	0	1	0	1	1	1	0	0	0	3	10	6	12
New start codon		5	138	21	1	0	2	6	1	2	1	2	2	1	1	1	1	3	2	3	1	0	3
New stop cocon		3	30	3	1	2	7	1	0	0	0	0	0	0	0	0	0	0	0	0	0	1	6

[1]Kugita *et al*., 2003; [2]Oldenkott *et al*., 2014; [3]Wolf *et al*., 2004; [4]Chen *et al*., 2011; [5]Wakasugi *et al*., 1996; [6]He *et al*., 2016; [7]Hein *et al*., 2016; [8]Lutz and Maliga, 2001; [9]Ruwe *et al*., 2013; [10]Hirose *et al*., 1999; [11]Inada *et al*., 2004; [12]Schmitz-Linneweber *et al.*, 2002; [13]Kahlau *et al.*, 2005; [14]Jiang *et al.*, 2012; [15]Lin *et al*., 2015; [16]Maier *et al*., 1995; [17]Corneille *et al.*, 2000; [18]Huang *et al*., 2013; [19]Calsa Junior *et al*., 2004; [20]Wang *et al.*, 2015; [21]Uthaipaisanwong *et al.*, 2012; [22]Lee *et al.*, 2014.

Supplementary Table 4. Plastid RNA edits in *rpo* transcripts among 18 species of higher plants.

Gene	Gene position	*Phalaenopsis aphrodite*	*Cycas taitungensis*	*Pinus thunbergii*	*Amborella trichopoda*	*Arabidopsis thaliana*	*Nicotiana tabacum*	*Pisum sativum*	*Atropa belladonna*	*Solanum lycopersicum*	*Ipomoea batata*	*Gossypium hirsutum*	*Zea mays*	*Oryza sativa*	*Cocos nucifera*	*Saccharum officinarum*	*Spirodela polyhiza*	*Elaeis guineensis*	*Deschampsia antarctica*
rpoA	200[a]	+/-	T	T	+	-	-	-	-	-	-	-	-	T	+	T	+	-	T
	368[a]	+/-	T	T	+	T	T	T	T	T	T	T	T	T	+	T	T	-	T
	527	-	T	T	+	T	T	T	T	T	T	T	T	-	+	T	T	-	+
	830[a]	+/-	+	T	T	T	+	T	+	+	T	-	T	T	+	T	T	-	T
rpoB	55[b]	+/-	-	-	-	-	-	-	-	-	-	-	-	-	-	-	-	-	-
	92[a]	+/-	-	T	+	-	-	-	-	-	-	-	-	-	-	-	T	-	-
	401[a]	+/-	T	T	T	T	+	T	+	+	-	+	T	T	T	T	T	T	T
	523	T	T	T	+	T	T	T	T	T	T	T	T	T	T	T	T	T	T
	536[a]	+/-	+	-	+	-	+	T	+	+	-	T	+	+	+	+	+	-	-
	614[a]	+/-	T	T	+	T	+	+	+	+	-	+	+	+	+	+	T	-	-
	629[a]	+/-	+	T	+	T	T	+	T	T	-	+	+	+	+	+	T	-	-
	686[a]	+/-	T	T	-	T	T	T	T	T	T	T	-	+	+	-	T	-	-
	1241	- (G)	T	T	+	T	T	T	T	T	T	T	T	T	T	T	T	T	T
	2038	T	-	T	+	T	T	T	T	T	T	T	T	T	T	T	T	T	T
	2063	T	T	T	T	T	+	+	+	+	-	T	T	T	+	T	T	-	T
	2489[a]	+/-	T	-	+	-	T	-	+	+	T	+	T	T	+	T	+	-	T
	2767	T	T	-	+	-	T	T	T	T	T	T	T	T	T	T	T	T	T
	2983	-	-	-	+	-	-	-	-	-	-	-	-	-	-	-	-	-	-
rpoC1	62[a]	+/-	+	T	T	T	+	+	+	+	-	+	-	-	+	-	+	-	T
	203[ab]	+/-	T	T	T	T	T	T	T	T	T	T	T	T	T	T	T	T	T
	243	-	T	T	+	T	T	T	T	T	T	T	T	T	T	T	T	T	T
	509[a]	+/-	-	-	+	-	T	-	T	T	T	-	T	T	T	T	-	-	T
	532	T	T	T	+	T	T	T	T	T	T	T	-	-	+	T	T	-	T
	573	-	T	-	+	T	-	-	-	-	-	-	T	T	-	T	T	-	T
	638[a]	+/-	T	T	-	T	T	T	T	T	T	T	T	T	+	T	-	-	T
	656	-	T	T	+	T	T	T	T	T	T	T	T	T	T	T	-	T	T
	808	T	T	T	+	T	T	T	T	T	T	T	T	T	T	T	T	T	T
	881	T	T	T	+	T	T	T	T	T	T	T	T	T	T	T	T	T	T
	947	T	T	T	+	T	T	T	T	T	T	T	T	T	T	T	T	T	T
	980	T	T	T	+	T	T	T	T	T	T	T	T	T	T	T	T	T	T
	1468	T	T	T	+	T	T	T	T	T	T	T	T	T	T	T	T	T	T
	1638[b]	+/-	T	-	-	T	-	T	-	-	-	-	T	T	-	T	T	-	T
	1927	T	T	T	+	-	T	T	T	T	T	T	T	T	T	T	T	T	T
rpoC2	548	T	T	T	+	T	T	T	T	T	T	T	T	T	T	T	T	T	T
	2336	T	T	-	+	T	T	T	T	T	T	T	+	T	-	+	+	-	-
	2681	-	-	-	-	-	-	T	-	-	T	-	T	-	-	T	-	-	-
	2693	-	T	T	-	-	-	-	-	-	-	-	-	T	-	-	-	-	-
	2846[a]	+/-	-	T	+	T	-	T	-	-	-	T	-	-	-	-	-	-	-
	3737	T	T	-	+	T	T	T	+	+	-	T	T	-	+	-	-	T	-
	4084	-	-	T	T	T	T	T	T	T	T	T	T	T	T	T	T	T	T

+ indicate editing; - indicate no editing; T indicate T at the DNA level (no editing at RNA level); +/- indicate partial editing (<90%) in orchid.

[a]Previously reported in Zeng *et al*., 2007; [b]Unique editing sites in moth orchid.

Supplementary Table S5. Comparative analysis of plastid RNA edits from flower tissue by two different bioinformatic approaches.

Gene[ψ]	Genome position*	Editing type	Edited ratio (%)		
			CLC Genomics Workbench	RES-Scanner with mq39[a]	RES-Scanner with mq30[b]
matK	1995	C→U	94.1	93.8	93.8
matK	2343	C→U	86.3	85.5	85.7
matK	2528	C→U	74.3	77.7	77.7
rps16-matK	3095	C→U	91.8	91.2	91.2
rps16	5026	C→U	90.3	85.3	89.3
rps25-psbK	6898	C→U	6.3	-	-
psbK-psbI	7837	C→U	13.4	15.5	16.6
atpA	11292	C→U	83.9	91.3	88.5
atpA	11667	C→U	94.8	95.0	95.1
atpF	13962	C→U	84.0	84.7	86.1
atpI	15279	C→U	97.6	97.5	97.7
atpI	15480	C→U	99.5	99.3	99.3
rps2	16782	C→U	90.8	91.2	91.8
rpoC2	18443	C→U	37.6	45.7	45.7
rpoC1	21899	C→U	10.3	8.2	8.2
rpoC1	22899	C→U	78.1	79.1	80.2
rpoC1	23028	C→U	84.8	83.3	85.3
rpoC1	24092	C→U	81.7	86.1	84.4
rpoC1	24233	C→U	72.2	74.2	74.2
rpoB	25087	C→U	76.5	73.3	74.2
rpoB	26890	C→U	66.3	73.2	74.0
rpoB	26947	C→U	68.3	79.5	79.5
rpoB	26962	C→U	72.2	82.1	82.8
rpoB	27040	C→U	71.1	81.1	76.8
rpoB	27175	C→U	76.4	79.2	70.4
rpoB	27484	C→U	55.4	53.1	53.1
rpoB	27521	C→U	10.4	11.5	11.5
psbM-rpoB	27736	C→U	30.9	33.3	33.3

trnD(gac)-psbM	31129	C→U	51.3	53.4	53.3
psbC	36287	C→U	13.4	14.9	14.6
trnG(gcc)-trnS(gga)	38003	C→U	7.7	-	-
rps14	38459	C→U	93.5	95.1	95.1
psaB	39498	C→U	7.2	-	-
ycf3-psaA	43763	C→U	-	6.7	8.5
ycf3 intron2	44389	C→U	47.8	50.7	51.4
ycf3	44880	C→U	81.8	72.5	83.5
ycf3	44886	C→U	94.9	87.2	94.8
ycf3 intron1	45108	C→U	86.4	90.0	90.2
ycf3	45758	C→U	93.2	89.3	94.2
trnT(aca)-rps4	47504	C→U	7.7	-	-
ndhJ-trnT(ugu)	49716	C→U	40.0	57.1	36.4
trnM(cau)	52826	C→U	63.6	71.9	73.5
atpB	53785	C→U	97.6	97.3	97.1
atpE-atpB	55019	C→U	-	6.8	6.7
rbcL-accD	57773	C→U	46.2	53.3	53.3
accD	58734	C→U	15.7	22.3	22.1
accD	59414	C→U	96.4	96.3	96.3
accD	59642	C→U	96.9	96.1	96.1
accD	59660	C→U	84.9	90.3	90.3
accD-psaI	59761	C→U	45.5	46.2	44.3
psaI	60705	C→U	92.3	92.4	92.7
psaI-ycf4	60754	C→U	-	7.5	7.3
psaI-ycf4	60764	C→U	59.2	62.8	62.3
psaI-ycf4	60775	C→U	-	5.6	5.5
psaI-ycf4	60946	C→U	-	-	5.1
ycf4	61295	C→U	5.1	5.3	6.3
cemA-petA	62818	C→U	14.9	17.1	16.7
psbJ	64925	C→U	17.4	19.4	19.3
psbF	65322	C→U	83.3	92.0	92.1
psbE	65604	C→U	7.1	7.4	8.1
petL	66862	C→U	46.8	65.3	65.3
petL	66913	C→U	84.7	86.1	86.3
petL-petG	67109	C→U	9.5	8.9	8.6
petG	67192	C→U	64.1	65.1	65.2

psaJ	68176	C→U	8.3	9.9	9.8
psaJ-rpl33	68296	C→U	20.4	21.6	21.6
rpl20	69674	C→U	9.0	10.3	10.0
rpl20	69969	C→U	13.3	14.2	14.1
clpP-rpl20	70010	C→U	26.8	28.7	27.9
clpP-rpl20	70142	C→U	90.8	90.7	90.4
clpP-rpl20	70726	C→U	23.9	22.2	22.9
clpP	71089	C→U	84.5	80.2	83.2
clpP	72255	C→U	92.0	92.2	93.0
clpP intron1	72384	C→U	11.0	9.4	10.4
psbB	74606	C→U	-	5.4	5.4
psbB-psbT	75495	C→U	58.3	61.5	61.5
psbN	76048	C→U	8.1	10.1	10.5
psbN	76049	C→U	7.9	10.1	10.6
psbN-psbH	76156	C→U	92.7	93.9	94.2
petB	77940	C→U	96.8	96.4	96.4
rpoA	79886	C→U	76.2	75.0	75.4
rpoA	80348	C→U	85.4	83.8	83.9
rpoA	80516	C→U	78.9	81.2	81.2
rps8	82172	C→U	88.8	89.7	90.0
rpl16 intron1	84617	C→U	9.8	9.2	8.8
rps3	84993	C→U	72.2	73.6	71.4
rps3	85546	C→U	5.3	7.2	7.1
rpl22	85813	C→U	6.5	7.5	7.1
petD-trnH(gug)	85997/148925*	U→C	12.6	NA	NA
rps19-rpl22	86046/148876*	C→U	9.8	NA	NA
rpl2-rps19	86586/148336*	C→U	22.7	NA	NA
rpl2	88243/146679*	C→U	71.0	NA	NA
rpl23	88474/146448*	C→U	88.4	NA	NA
trnH(gug)-ycf2	88550/146372*	C→U	7.9	NA	NA
trnH(gug)-ycf2	88588/146334*	C→U	5.3	NA	NA
ycf2	90914/144008*	C→U	6.5	NA	NA
ycf2-trnV(gac)	95813/139109*	C→U	7.1	NA	NA
ndhB$^{\psi}$	97389/137533*	C→U	56.8	NA	NA
ndhB$^{\psi}$	99260/135662*	C→U	9.9	NA	NA
rps12 intron1	100465/134457*	C→U	5.4	NA	NA

rps12 intron1	100611/134311*	C→U	54.4	NA	NA
rps12	100922/134000*	C→U	89.7	NA	NA
trnN(guu)-rps12	104082/130840*	C→U	8.1	NA	NA
trnR(acg)-rpl32	111392/123530*	C→U	6.7	NA	NA
trnR(acg)-ycf1	111623/123299*	C→U	8.7	NA	NA
rpl32	112003	C→U	13.9	6.6	5.9
ccsA	113255	C→U	9.0	11.3	11.0
ccsA	113571	C→U	-	5.2	5.1
ndhE[Ψ]	116438	C→U	17.3	19.3	18.9
ccsA-trnN(guu)	117201	C→U	8.6	10.3	10.3
rps15	117323	C→U	14.2	16.7	16.7
ycf1	120628	U→C	-	-	12.8
ycf1	120647	U→C	-	8.7	17.6
ycf1	121075	U→C	-	8.2	7.5
ycf1	121110	C→U	-	18.2	18.2
ycf1	121980	C→U	8.7	17.4	16.0
ycf1	123161	U→C	-	-	37.5
ccsA-trnN(guu)	123231	C→U	6.9	-	-

[*]Indicated that edits located in inverted repeat region

[Ψ]Indicated that *ndh* genes are truncated pseudogenes in moth orchid.

[a]Indicated that Phred-scaled base quality score cutoff 30 and mapping quality cutoff 39 used in RES-scanner.

[b]Indicated that Phred-scaled base quality score cutoff 30 and mapping quality cutoff 30 used in RES-scanner.

[-]Indicated that no editing or editing ratio <5%.

[NA]Indicated that edits located in inverted repeat regions which it can not be analyzed by RES-scanner.

Supplementary Fig. 1

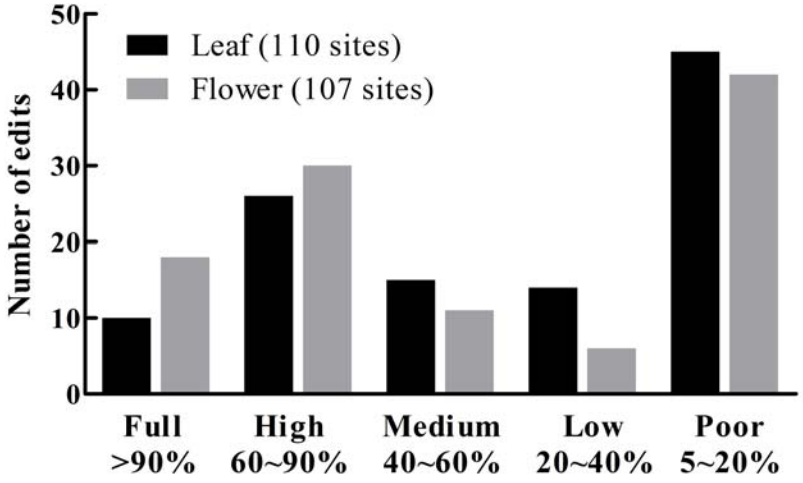

Supplementary Fig. 1. The editing efficiency of plastid RNA edits in leaf and floral tissues.

According to the edited percentage for each nucleotide in sequence reads mapping to the corresponding cpDNA, the efficiency of RNA editing for each site was classified into five groups: fully edited (>90%), high partially edited (60~90%), medium partially edited (40~60%), low partially edited (20~40%) and poor partially edited (5~20%). The number in leaf (black bar) and floral tissue (gray bar) is shown.

Supplementary Fig. 2

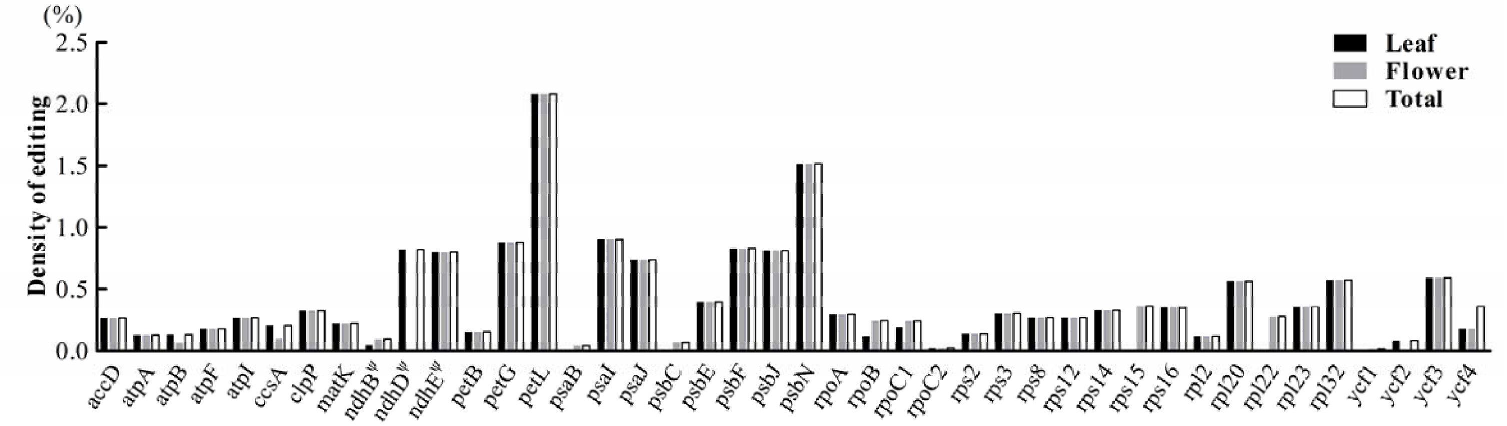

Supplementary Fig. 2. The density of RNA editing sites in protein-coding transcripts.

The density of RNA edits for each transcript of plastid protein-coding genes in leaf (grey), floral (black), or both (white) tissues is shown. The editing density was estimated by number of RNA editing sites in each protein-coding transcript (Supplemental Table 1) divided by the length (kb) of the corresponding transcript.

Supplementary Fig. 3

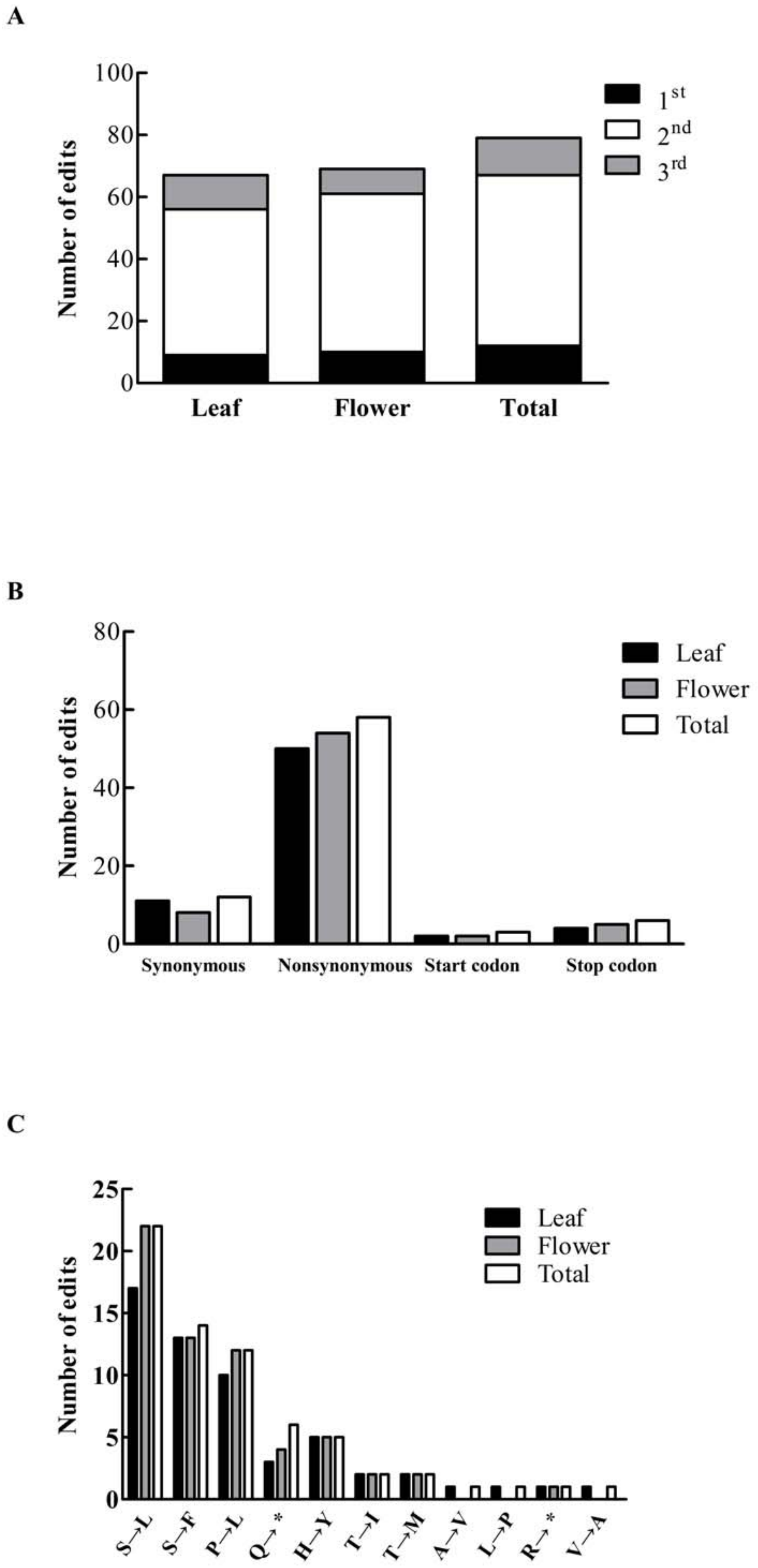

D

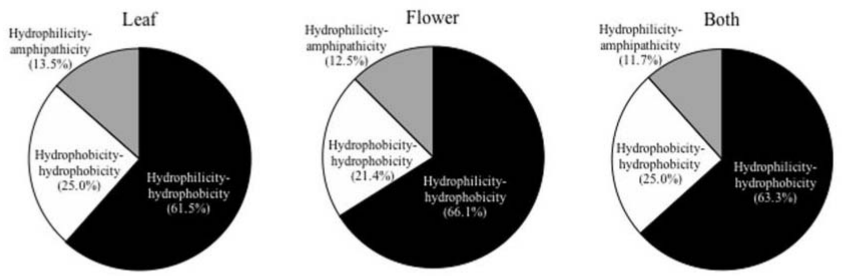

Supplementary Fig. 3 RNA editing in protein-coding transcripts.

(A) The number of edited codon positions for protein-coding transcripts in leaf, floral and both tissues is shown. (B). The number of RNA editing resulted in synonymous substitution, non-synonymous substitution, creation of start and stop codons in leaf (black bar), flower (gray) and both tissues (white) is shown. (C). The distribution of amino acid changes resulting from a C-to-U conversion in leaf (black bar), flower (gray) and both tissues (white) is shown. The asterisk indicates stop codon. (D). The percentage of amino acid property changed after RNA editing in leaf (left), flower (middle) and both tissues (right).

Supplementary Fig. 4

A.

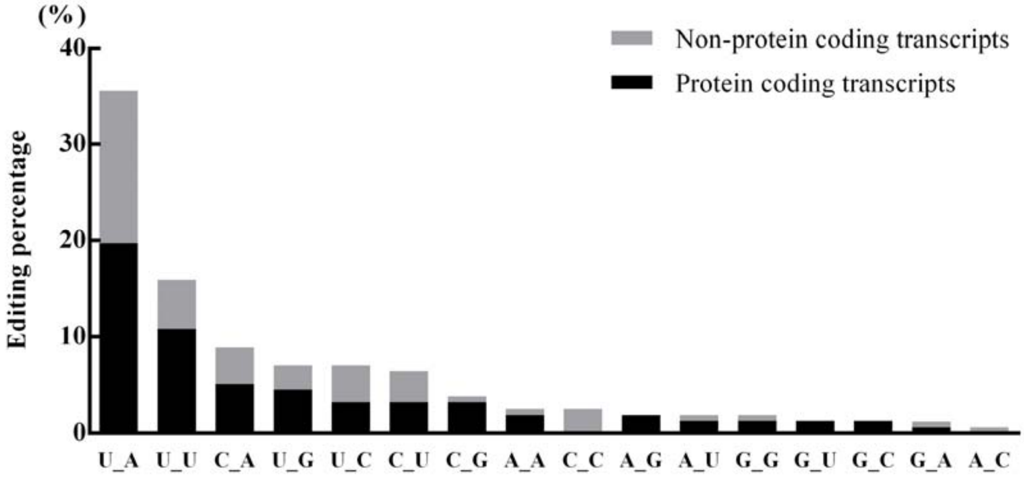

B.

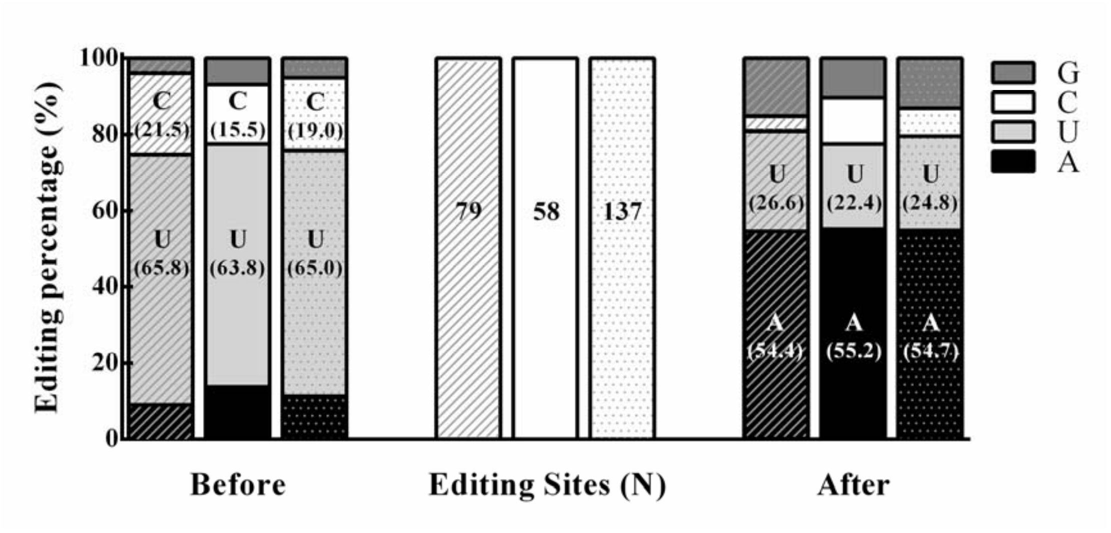

Supplementary Fig. 4 Nearest-neighbor bias toward a U_A context immediately before and after plastid RNA edits in moth orchid.

(A). The percentage indicates the ratio and distribution of specific nucleotides immediately before or after editing sites in protein (black bar) and non-protein (grey bar) coding transcripts, respectively, over the total number (137) of RNA edits. (B). Nearest-neighbor bias toward a U_A context immediately before and after edits in protein-coding transcripts (N=79), non-protein-coding transcripts (N=58) and total transcripts (N=137).

Supplementary Fig. 5

(A) *rps12* intron (Genome position: 100,611)

Unedited

Secondary structure: ΔG = −18.4kcal/mol

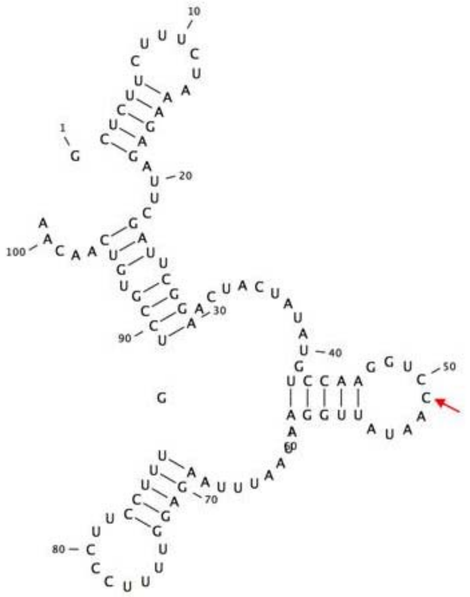

Edited

Secondary structure: ΔG = −18.4kcal/mol

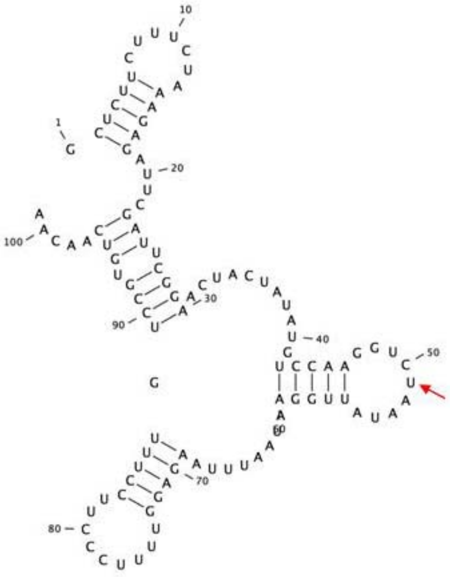

(B) *ycf3* intron (Genome position: 45,108)

Unedited

Secondary structure: ΔG = −21.7kcal/mol

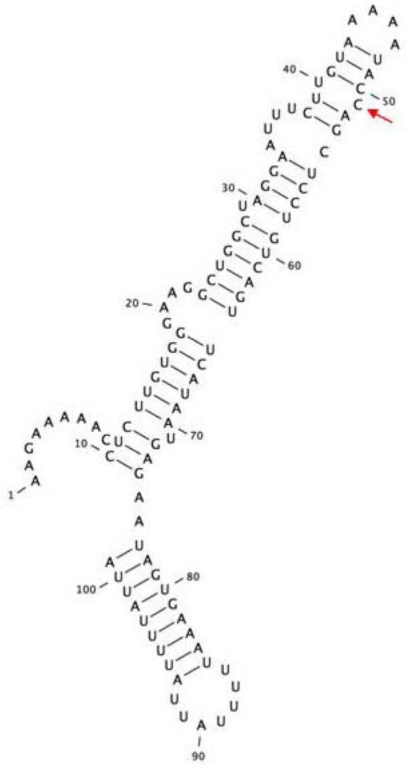

Edited

Secondary structure: ΔG = −21.7kcal/mol

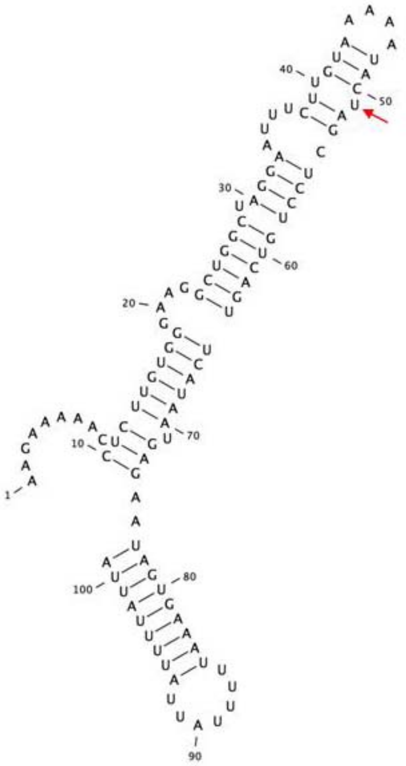

(C) *ycf3* intron (Genome position: 44,389)

Unedited

Secondary structure: ΔG = −11.7kcal/mol

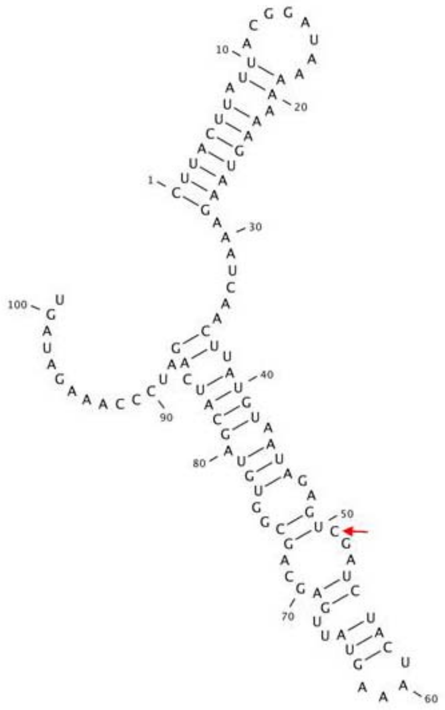

Edited

Secondary structure: ΔG = −15.6kcal/mol

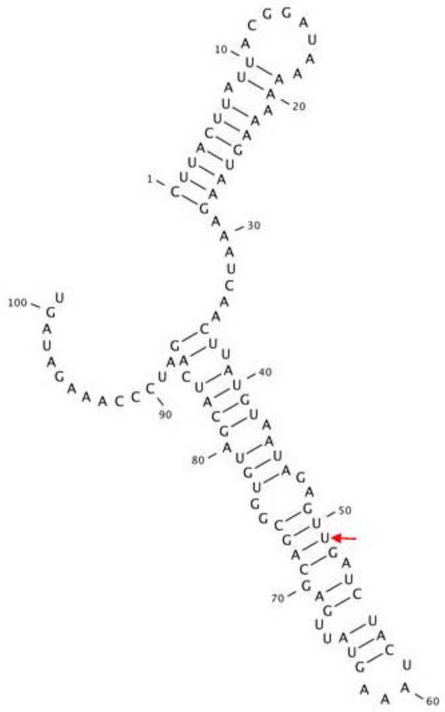

(D) *psaI-ycf4* (Genome position: 60764)

Unedited

Secondary structure: ΔG = −13.4kcal/mol

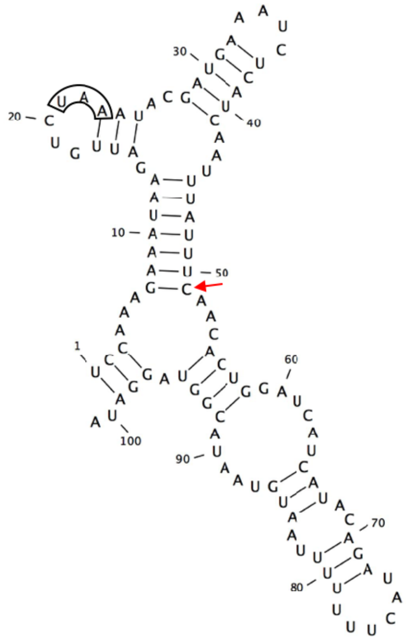

Edited

Secondary structure: ΔG = −10.9kcal/mol

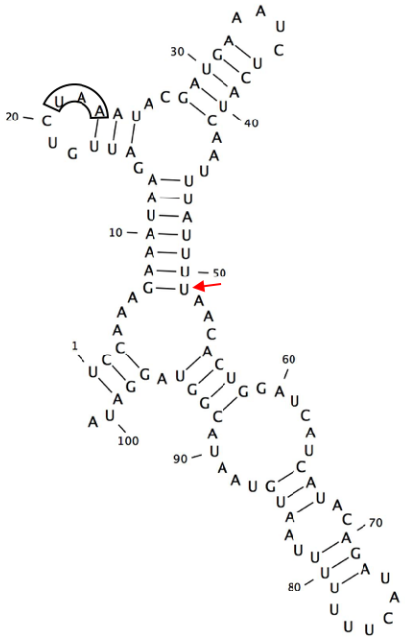

(E) *rps16-matK* (Genome position: 3095)

Unedited

Secondary structure: ΔG = -13.2kcal/mol

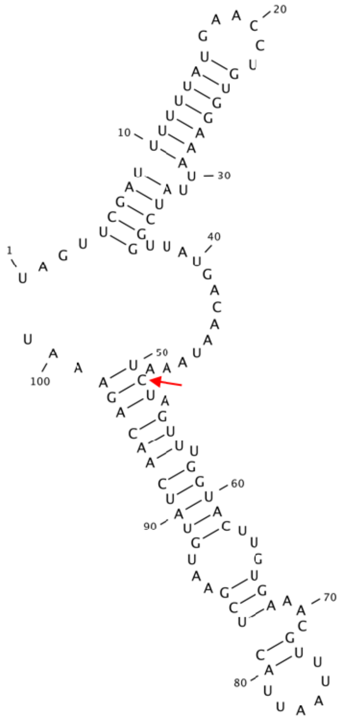

Edited

Secondary structure: ΔG = -11.8kcal/mol

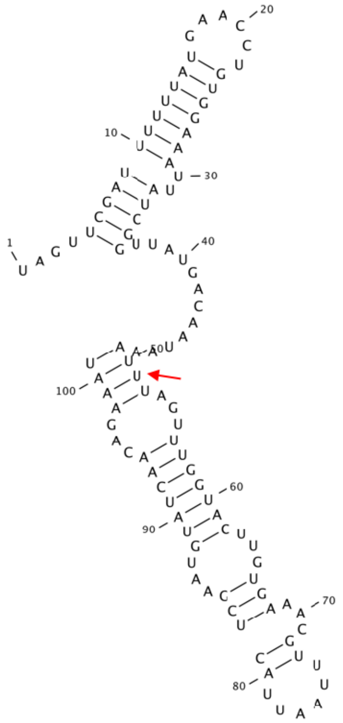

(F) *clpP-rpl20* (Genome position: 70142)

Unedited

Secondary structure: ΔG = -17.3kcal/mol

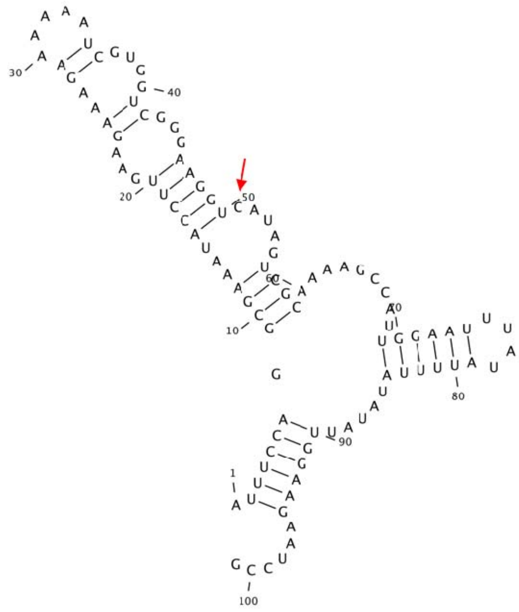

Edited

Secondary structure: ΔG = -18.0kcal/mol

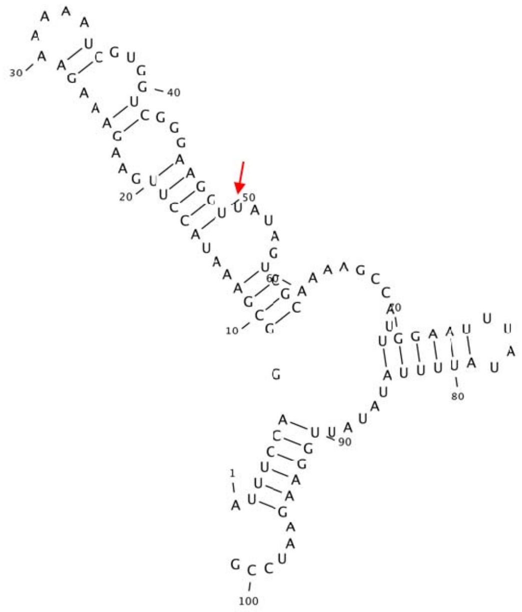

(G) *psbM-rpoB* (Genome position: 27736)

Unedited

Secondary structure: ΔG = −9.3kcal/mol

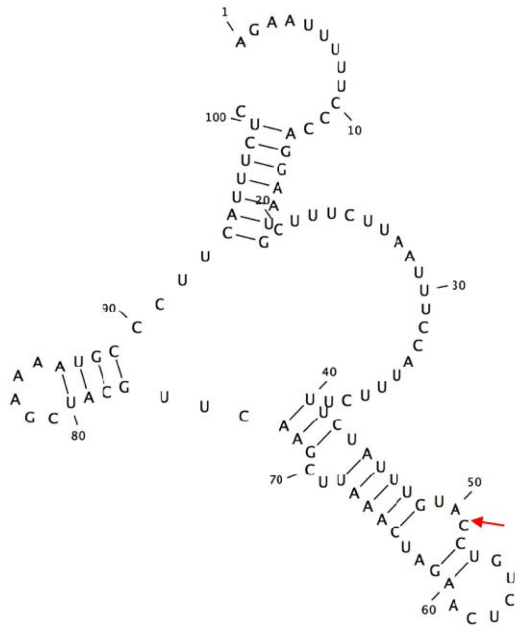

Edited

Secondary structure: ΔG = −13.0kcal/mol

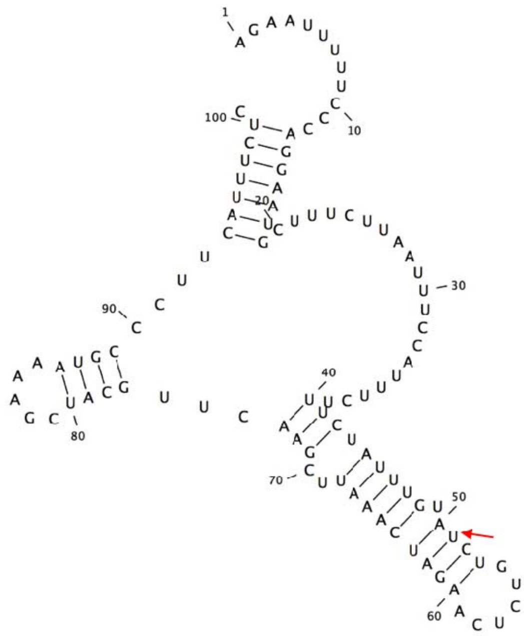

(H) *trnD-pabM* (Genome position: 31129)

Unedited

Secondary structure: ΔG = −14.7kcal/mol

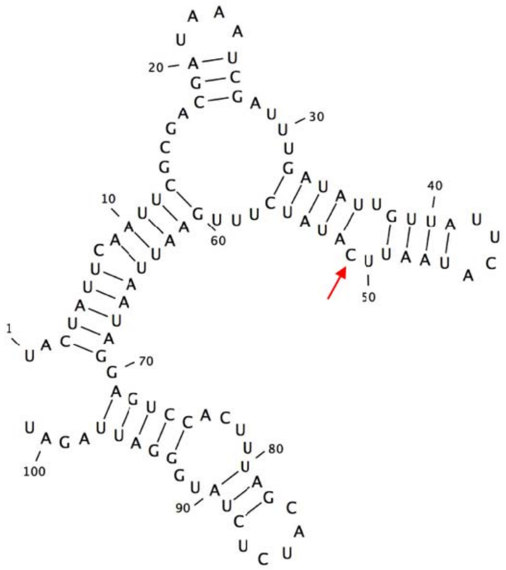

Edited

Secondary structure: ΔG = −15.2kcal/mol

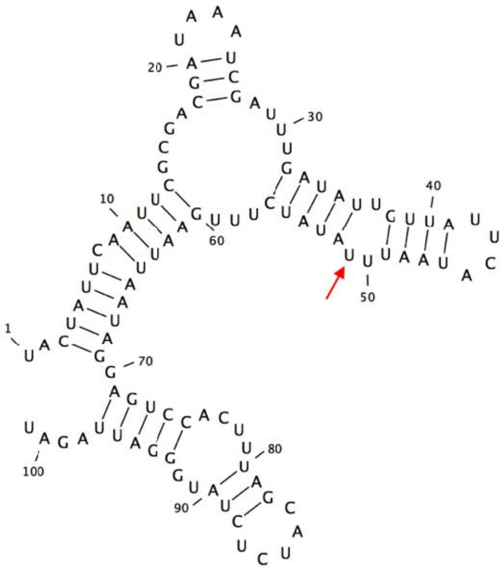

(I) *psbB-psbT*(Genome position: 75495)

Unedited

Secondary structure: ΔG = −12.4kcal/mol

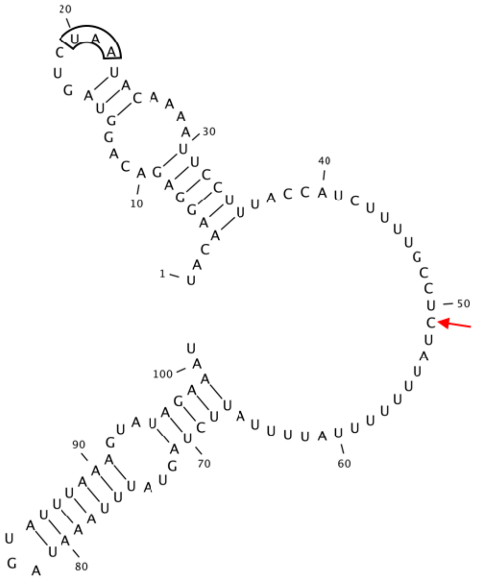

Edited

Secondary structure: ΔG = −12.4kcal/mol

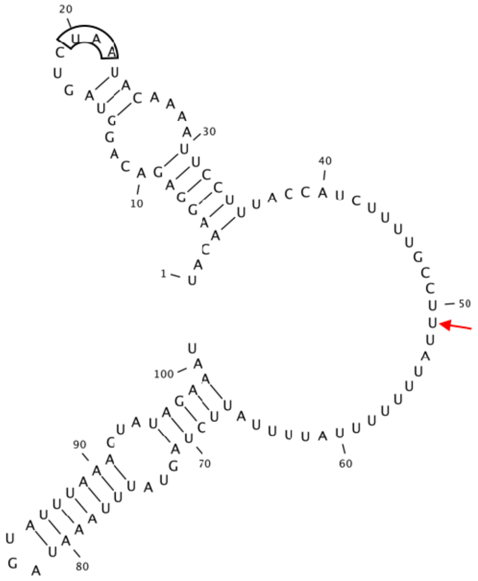

(J) *trnN-rps12*(Genome position: 130345)

Unedited

Secondary structure: ΔG = −24.2kcal/mol

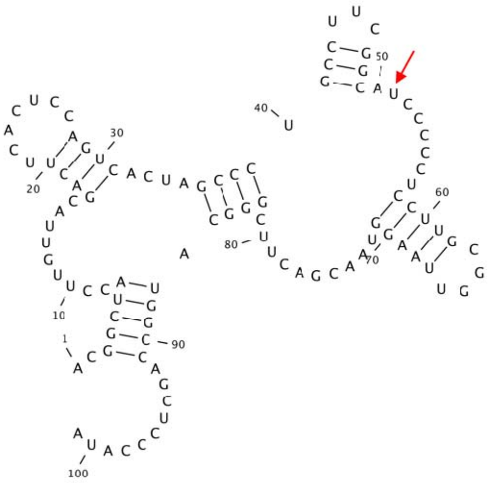

Edited

Secondary structure: ΔG = −24.2kcal/mol

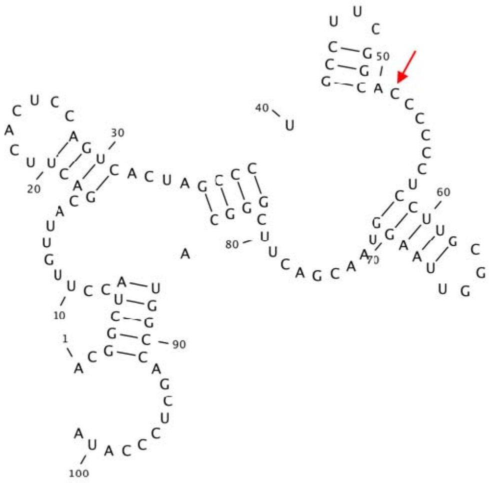

Supplementary Fig. 5 The prediction of RNA secondary structures formed by the unedited and edited plastid transcripts in moth orchid.

The predicted RNA secondary structures formed by the unedited and edited transcripts in the intron and intergenic spacer regions by using CLC genomic Workbench. The edited and unedited nucleotides are indicated by red arrows. (A). *rps12* intron (100,611); (B). *ycf3* intron (45,108); (C). *ycf3* intron (44,389); (D). *psaI-ycf4* (60,764); (E). *rps16-matK* (3,095); (F). *clpP-rpl20* (70,142); (G). *psbM-rpoB* (27,736); (H). *trnD(gac)-psbM* (31129); (I). *psbB-psbT* (75,495); (J). *trnN-rps12*(130345).
